# Deep learning-enabled realistic virtual histology with ultraviolet photoacoustic remote sensing microscopy

**DOI:** 10.1038/s41467-023-41574-2

**Published:** 2023-09-25

**Authors:** Matthew T. Martell, Nathaniel J. M. Haven, Brendyn D. Cikaluk, Brendon S. Restall, Ewan A. McAlister, Rohan Mittal, Benjamin A. Adam, Nadia Giannakopoulos, Lashan Peiris, Sveta Silverman, Jean Deschenes, Xingyu Li, Roger J. Zemp

**Affiliations:** 1 Department of Electrical and Computer Engineering, University of Alberta, 116 Street & 85 Avenue, Edmonton, AB T6G 2R3 Canada; 2 Department of Laboratory Medicine and Pathology, University of Alberta, 11405 87 Avenue NW, Edmonton, AB T6G 1C9 Canada; 3 Department of Surgery, University of Alberta, 8440 - 112 Street, Edmonton, AB T6G 2B7 Canada

**Keywords:** Surgical oncology, Optical imaging, Molecular imaging, Cancer imaging, Breast cancer

## Abstract

The goal of oncologic surgeries is complete tumor resection, yet positive margins are frequently found postoperatively using gold standard H&E-stained histology methods. Frozen section analysis is sometimes performed for rapid intraoperative margin evaluation, albeit with known inaccuracies. Here, we introduce a label-free histological imaging method based on an ultraviolet photoacoustic remote sensing and scattering microscope, combined with unsupervised deep learning using a cycle-consistent generative adversarial network for realistic virtual staining. Unstained tissues are scanned at rates of up to 7 mins/cm^2^, at resolution equivalent to 400x digital histopathology. Quantitative validation suggests strong concordance with conventional histology in benign and malignant prostate and breast tissues. In diagnostic utility studies we demonstrate a mean sensitivity and specificity of 0.96 and 0.91 in breast specimens, and respectively 0.87 and 0.94 in prostate specimens. We also find virtual stain quality is preferred (*P* = 0.03) compared to frozen section analysis in a blinded survey of pathologists.

## Abstract (web summary)

Oncologic tumour resection is not fully accurate. Here the authors report a label-free virtual histological imaging method based on a non-contact, reflection-mode ultraviolet photoacoustic remote sensing and scattering microscope, combined with unsupervised deep learning using a cycle-consistent GAN.

## Introduction

Surgical resection is the primary treatment for many solid tumors. In oncologic surgeries, surgeons attempt to excise malignant tissue along with a surrounding clear margin, while sparing as much healthy tissue as possible. The margins of a resected specimen are then evaluated for involvement of malignant cells, ensuring that the primary cancer has been completely removed. The status of surgical margins is therefore considered one of the strongest indicators of operative success, and long-term patient prognosis. Unfortunately, gross visual and tactile inspection of specimens offers limited sensitivity for delineating boundaries between healthy and malignant tissues, allowing microscopic pathology to go undetected. Positive margins are still found under postoperative analysis in up to 40% of cases^1^ depending on the type of tumor, often necessitating follow-up re-excision surgery, and adjuvant chemotherapy, radiation, or endocrine therapies. The problem of positive margins not only causes additional physical and emotional trauma for patients and leads to increased morbidity, but also results in delays, excess expenses, and consumption of limited resources within the healthcare system^2^. The development of improved microscopic imaging methods for intraoperative margin status assessment could significantly enhance patient care and contribute to a more cost-efficient healthcare economy.

**Fig. 1:**
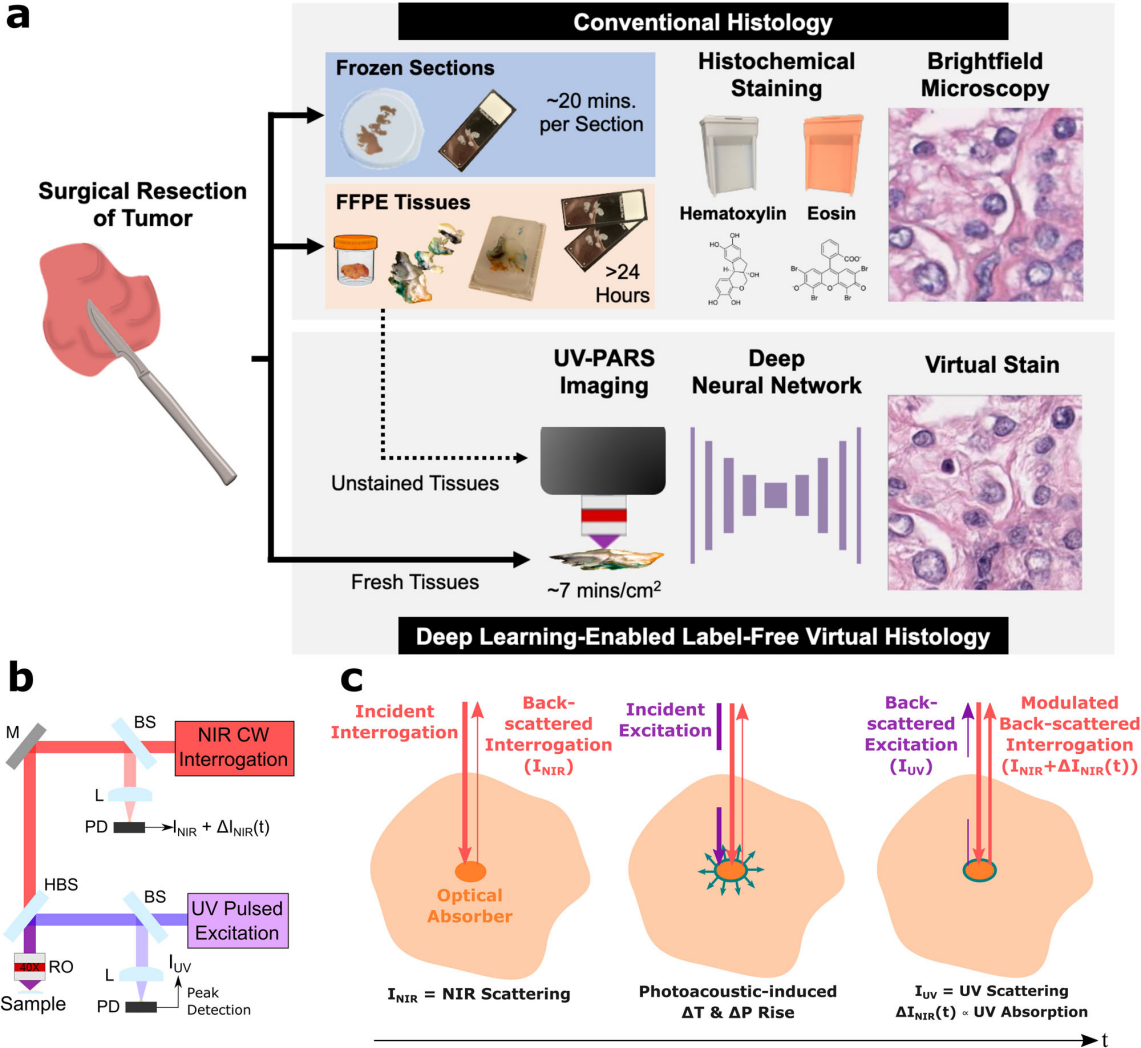
Comparison of proposed UV-PARS virtual histology and gold standard histology workflows. **a** Conventional surgery-to-histopathology workflow (top) comparing frozen sections to FFPE tissue methodologies, in contrast to our deep learning-enabled label-free histology approach (bottom) that is capable of bypassing tissue preparation and/or staining steps to rapidly obtain virtual histology. **b** A simplified system diagram of our combined UV-PARS and UV scattering microscopy system capable of providing simultaneous absorption and scattering contrast, respectively. M mirror, L lens, BS beamsplitter, HBS harmonic beamsplitter, RO reflective objective, PD photodiode. **c** Visualization of the PARS mechanism.

The current gold standard for margin assessment involves pathologist inspection of hematoxylin and eosin (H&E) stained thin sections of tissues under brightfield microscopy. This allows identification of atypical cytologic features including an increased nucleus-to-cytoplasm ratio, variations in the size and shape of cells and nuclei, and increased or abnormal mitosis, in addition to various architectural patterns which differentiate benign and malignant tissues. However, preparation of 4–5 μm thin formalin-fixed paraffin-embedded (FFPE) tissue sections compatible with brightfield light microscopy involves many time-consuming and labor-intensive steps (Fig. 1a), typically resulting in minimum turnaround times of nearly 24 h. FFPE H&E histopathology is therefore unsuitable for providing intraoperative feedback.

Conventional alternatives to FFPE H&E histopathology exist to offer rapid assessment of margin status while the patient remains in the surgical suite, though they suffer from limitations in technical quality and diagnostic accuracy in some applications^3–7^. Frozen section analysis (FSA) can generate slides available for brightfield microscopy in approximately 20 mins, which is considerably faster than FFPE permanent sectioning. However, drawbacks include errors due to limited sampling of the tissue, a high dependence on operator skill, and the destructive nature of the tissue preparation process, which may leave insufficient tissue for downstream histology, molecular assays, or genetic analysis to provide a definitive diagnosis. Frozen sections are frequently subject to significant freezing artifacts, which can lead to diagnostic inaccuracies^6^. Moreover, lipid-rich tissues such as breast do not freeze well and are difficult to cut into thin sections. FSA is consequently not performed routinely in resection surgeries for the majority of tumor types. Touch imprint cytology is another fast alternative to FFPE sectioning, though it also suffers from known performance limitations^8^.

Recent efforts have developed methods capable of virtual histological imaging. These methods aim to bypass much of the conventional tissue processing to rapidly generate realistic H&E-like virtual imagery that can be directly interpreted by pathologists, making diagnostic feedback available earlier in the standard histopathology workflow (Fig. 1a). In this study, we introduce a rapid, high-resolution virtual histology method which achieves histological realism in unstained tissues with demonstrated advantages over FSA in a blinded pathologist study, and exhibits strong quantitative similarity and diagnostic concordance with true H&E-stained brightfield histology in both breast and prostate tissues.

In brief, our virtual histology approach uses ultraviolet photoacoustic remote sensing (UV-PARS) microscopy to achieve hematoxylin-like contrast to visualize cell nuclei, and ultraviolet scattering microscopy to provide eosin-like contrast for imaging cytoplasm and the extracellular matrix (Fig. 1b). PARS is a non-contact laser scanning photoacoustic imaging modality which detects intensity modulations in a back-scattered interrogation beam induced by optical absorption of a nanosecond-pulsed excitation beam^9–11^ as shown in Fig. 1c. UV-PARS was first demonstrated by Haven et al.^12^ for cell nuclei imaging and further improved in subsequent reports^13,14^. Integration of near-infrared (NIR) scattering microscopy further enabled simultaneous acquisition of complementary virtual eosin contrast co-registered with the UV-PARS images^15–17^, simplifying the complexity of previous dual-contrast approaches^18,19^. The NIR scattering was later upgraded to 266 nm pulsed UV scattering along with a pulse peak sample-and-hold detection circuit to achieve improved resolution in the virtual eosin data^20^ utilizing the existing excitation laser source. In this work we utilize a high-resolution 0.5 numerical aperture (NA) objective with rapid voice coil scanning capable of imaging at speeds of up to 7 mins/cm^2^ with 390 nm lateral resolution^21^, and axial optical sectioning of 1.6 μm similar to thin histology sections^22^. Compared to existing virtual histology techniques, where key specifications are outlined in Supplementary Table 1, our microscopy approach offers an advantageous combination of scan speed, resolution, histological realism with positive nuclear contrast, and label-free imaging capabilities.

Virtual histology methods based on fluorescent staining include light-sheet microscopy^23,24^, microscopy with ultraviolet surface excitation (MUSE)^25^, nonlinear microscopy^26,27^, and confocal fluorescence microscopy^28^. Though uptake of dyes such as acridine orange, proflavine, DAPI, propidium iodide, Hoechst, and rhodamine typically adds only up to a few minutes to the sample processing workflow, the use of exogenous dyes or fluorescent labels can be subject to staining variability affecting interpretation^29^. Compared to label-free approaches, the reliance on fluorescent dyes for initial virtual H&E histology may also interfere with subsequent special stains, immunohistochemistry, or fluorescence in situ hybridization of the same tissue section. Additionally, the toxicity and lack of FDA approval for certain agents may restrict their application to only fixed tissues, precluding future in vivo extensions. Volumetric imaging techniques such as light-sheet microscopy may also require optical clearing agents. While camera-based widefield imaging techniques such as MUSE and light-sheet microscopy offer remarkable imaging speed, the resulting resolution trade-off may be unacceptable to pathologists in some cases compared to the confocal sectioning of a laser-scanning microscopy approach. MUSE particularly benefits from a simplified, cost-effective implementation for slide-free imaging, though it relies on tissue-dependent UV penetration depth discrimination which may result in simultaneous detection of multiple cell layers, potentially obfuscating diagnostic interpretations as noted in several reports^30,31^.

Label-free virtual histology methods based on autofluorescence emission include simultaneous label-free autofluorescence-multiharmonic (SLAM) microscopy^32^, swept confocally-aligned planar excitation light-sheet microscopy (MediSCAPE)^24^, computational high-throughput autofluroescence microscopy by pattern illumination (CHAMP)^33^, and conventional transillumination autofluorescence microscopy^34^. Other mechanisms providing label-free contrast analogous to H&E staining include stimulated Raman scattering (SRS) based on CH_2_ and CH_3_ shifts^35,36^, multispectral deep-UV microscopy^37^, and deep learning-aided reflectance confocal microscopy (RCM)^38^. While optical coherence tomography (OCT) is a rapid label-free cross-sectional imaging technology with demonstrated clinical value, most implementations lack sub-cellular lateral resolution and molecular specificity to important cytologic details including nuclei, limiting utility for virtual histology^39^.

Ultraviolet photoacoustic microscopy (UV-PAM)^40–46^ provides a label-free approach for generating positive nuclei contrast through absorption-induced thermoelastic expansion. Cytoplasmic contrast can also be obtained via additional cytochrome-targeted excitation wavelengths^47^, or by detecting photoacoustic signals originating from relatively weaker absorption in the cytoplasm, using deep learning-assisted inference^46^. These methods however suffer from mismatched nuclear and cytoplasmic resolutions, and weak cytoplasmic signal-to-noise ratios, respectively. This may lead to morphological differences in the resulting virtual stain. Additionally, the requirement for acoustic coupling or immersion can limit scan speeds, and restrict reflection-mode imaging to lower NA focusing with lateral and axial resolutions sacrificed. In contrast, our lateral and axial resolutions of 390 nm and 1.6 μm, respectively, are finer than related UV-PAM methods. For instance, Cao et al.^48^ report a 40 μm acoustic axial resolution and 9 μm optical depth-of-focus, with 960 nm lateral resolution. A 330 nm lateral resolution imaging has been achieved in transmission-mode^41^, though this is not suitable for thick tissue imaging. Acoustic coupling additionally precludes the use of a coverslip for tissue flattening, necessitating slower contour scanning of the tissue surface for imaging thick specimens.

**Fig. 2:**
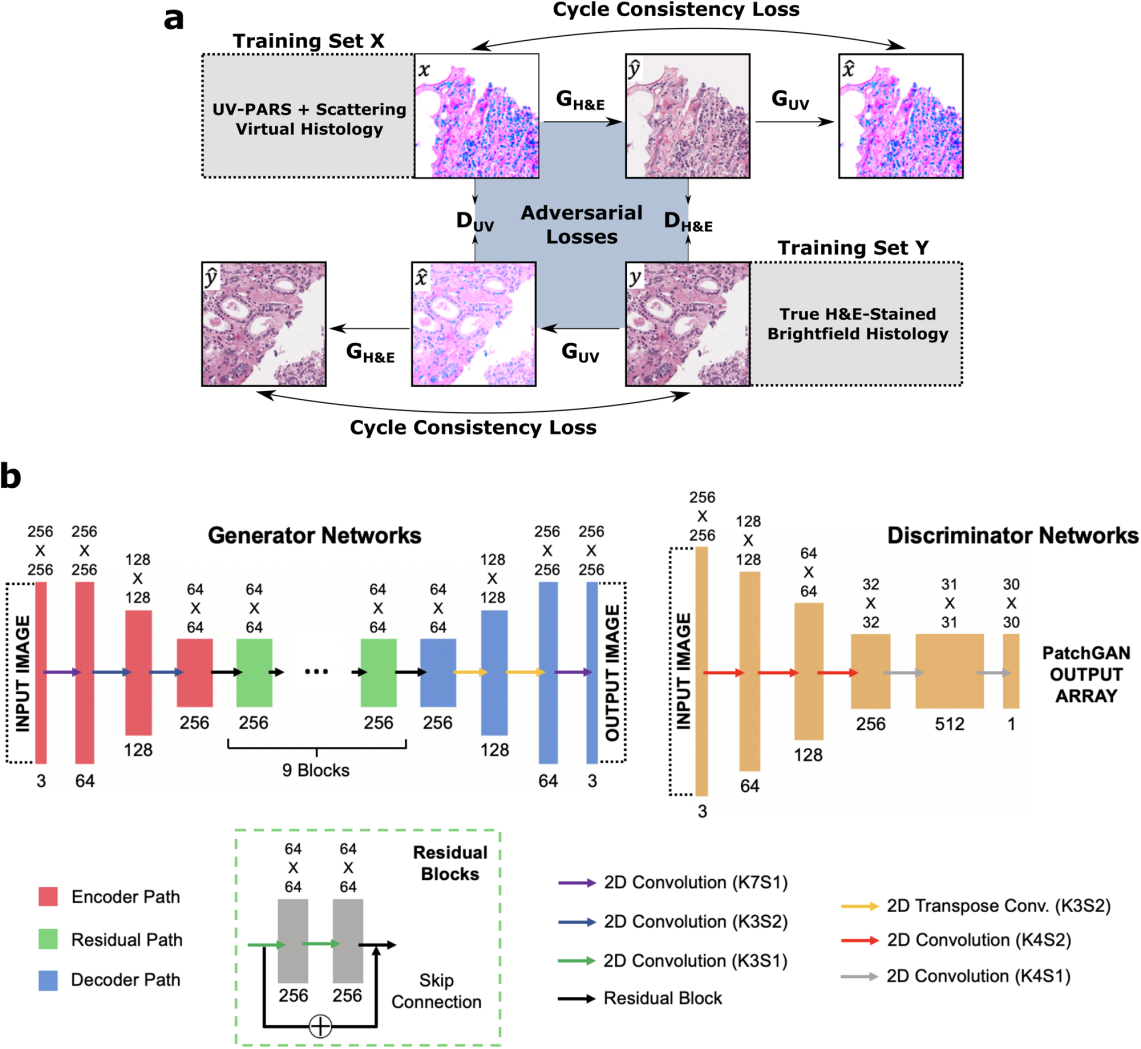
CycleGAN principle and deep neural network architectures. **a** Unsupervised training is performed with unpaired input data sets including virtual images *X* and true brightfield H&E-stained images *Y*. Forward and reverse generator transformations *G*_*H&E*_ and *G*_*UV*_ are trained concurrently with corresponding discriminators *D*_*H&E*_ and *D*_*UV*_, which progressively improve their ability to classify generated synthetic images from true input examples. Images used for each cycle consistency and adversarial loss function are indicated. **b** Detailed architectures of the constituent deep convolutional neural networks used in the CycleGAN model. K convolution kernel size, S convolution stride.

To render virtual histology in a realistic stain style comparable to gold standard H&E-stained brightfield microscopy, powerful deep learning methods have emerged^33,34,38,39,46,49,50^, offering unparalleled performance compared to alternatives such as lookup table-based pseudo-coloring or physics-based stain blending models^51^. In this work, we leverage the dual-contrast input image data from our UV-PARS microscope, with hematoxylin-like positive nuclei contrast and high-resolution eosin-like scattering contrast, combined with a cycle-consistent generative adversarial network (CycleGAN)^52^ to generate maximally-realistic virtual H&E histology. This will be important for stain normalization to reduce variability, and for applying existing computer-aided diagnosis algorithms developed for digital pathology to our virtual histology images, which would otherwise be incompatible. For image style transfer, a generative adversarial network (GAN) uses the concept of adversarial training, where a generator network constructs plausible new candidate images, and is trained in zero-sum competition with a discriminator network attempting to differentiate synthetic generator output images from presented examples of real image data^53^. With optimized training, the goal is to learn the underlying distribution of the target image domain. For virtual histology, conditional GAN^54^ approaches like the pix2pix model^55,56^ require supervised training with paired input data featuring matching tissue morphology, with precise co-registration necessary for optimal performance. In contrast, the CycleGAN algorithm concurrently trains both a forward and inverse model, exploiting invertibility to learn a distribution matching style transformation from the input modality to brightfield H&E-stained histology using unpaired training data sets (Fig. 2a). Cycle-consistency constraints further regularize the unsupervised training approach, ensuring that transforming an image to the opposing domain then reconstructing in the original domain achieves an image that is minimally different from the initial input in the L1 sense. The detailed architectures of the deep convolutional neural networks used in this study are outlined in Fig. 2b.

The CycleGAN approach is important in our context because it is often challenging or impossible to obtain virtual histology images with a closely-matching paired true H&E counterpart. This is particularly true for thick tissues and multi-layer imaging, where the fixing, embedding, sectioning, and staining processes will introduce morphological differences which would significantly degrade the performance of a supervised learning approach. Additionally, the CycleGAN is suitable for stain style matching to existing digital histology databases, and affords training of new style transfer models for numerous special stain types beyond H&E using the same virtual histology data set, without the complications of de-staining. This may also facilitate simplified integration into existing clinical workflows, as the virtual H&E stain style can be easily targeted to match individual pathologist preferences or standardized institutional protocols.

In this report, we present a deep learning-enabled, high-resolution, and label-free approach capable of producing maximally-realistic virtual histology with rapid acquisition times as low as 7 mins/cm^2^. We test our approach on FFPE breast and prostate tissues from human subjects and in freshly-resected thick murine liver and kidney tissues. Quantitative metrics are used to validate similarity between our virtual histology approach and the gold standard. Additionally, based on pathologist-rated stain quality metrics, our virtual histology images were found to offer superior nuclear detail and were preferred overall compared to FSA. Furthermore, initial diagnostic concordance studies in both breast and prostate tissues suggest strong diagnostic utility to pathologists. Results show that our approach has the potential to scan large en-face thick tissues within intraoperative time frames.

## Results

### Virtual staining of human breast and prostate tissue specimens

**Fig. 3:**
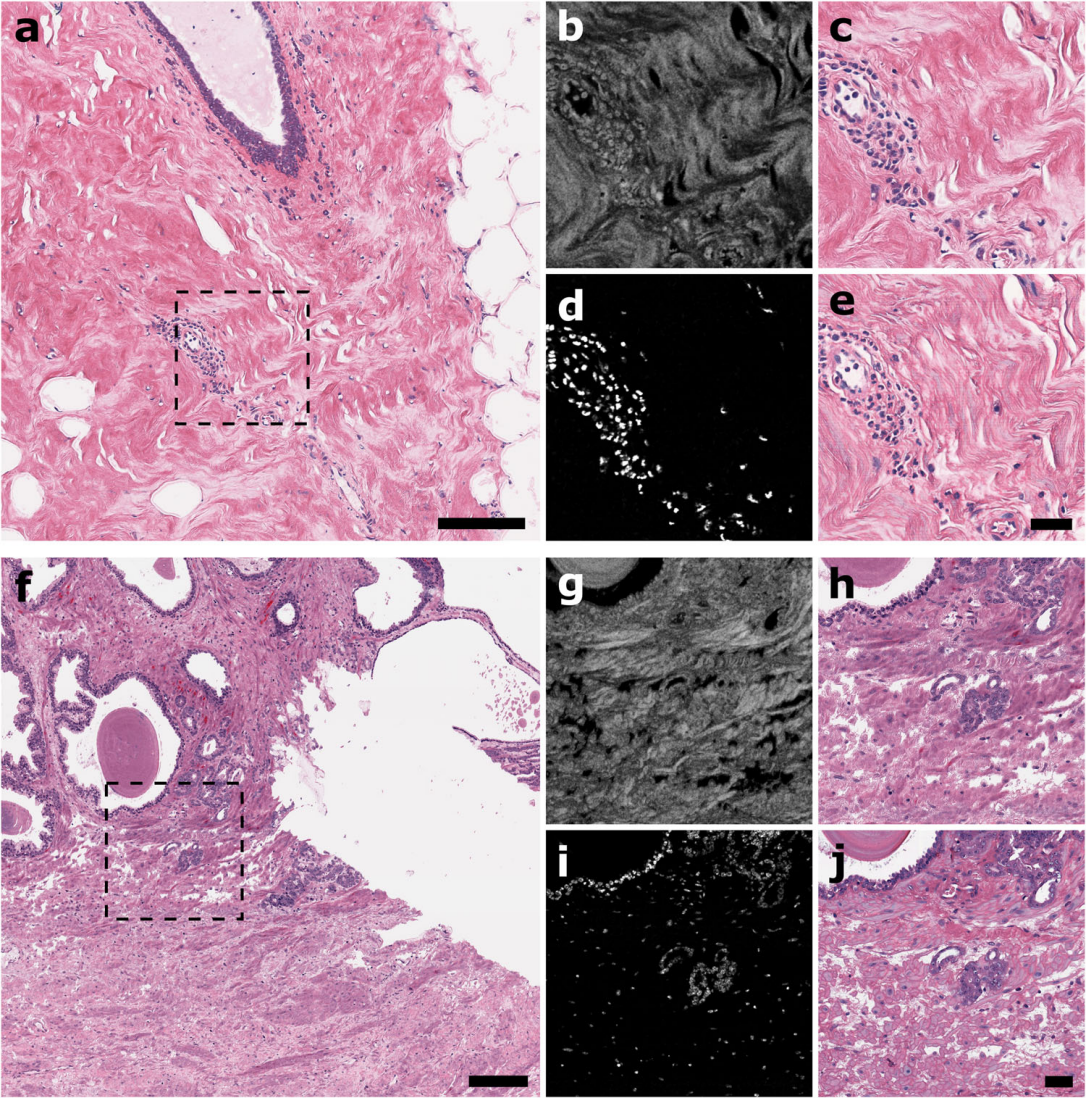
Deep learning-enabled realistic virtual histology. **a** Image of unstained breast tissue. **f** Image of unstained prostate tissue. Scale bars: 200 μm. **b** UV scattering channel data. **d** UV-PARS channel data, (**c**) virtual histology image, and (**e**) corresponding true H&E-stained brightfield histology image for dashed box inset in (**a**). Scale bar: 50 μm. **g** UV scattering channel data, (**i**) UV-PARS channel data, (**h**) virtual histology image, and (**j**) corresponding true H&E-stained brightfield histology image for dashed box inset in (**f**). Scale bar: 50 μm.

We tested our virtual histology approach on both sectioned and unstained human lumpectomy and radical prostatectomy specimens. UV-PARS and UV scattering data, unseen in the initial training set, were input into their respective trained CycleGAN networks and compared to true H&E brightfield microscopy images for validation. Figure 3a shows a deep learning-enabled virtual histology image of breast tissue. UV scattering and UV-PARS data channels for the area in the dashed box inset in (a) are shown in (b) and (d), respectively. Comparing the virtual histology inset in (c) to a true H&E-stained brightfield image in (e), the stromal structure and nuclear details are concordant, with the structure of a benign blood vessel being clearly identifiable by pathologists. Figure 3f shows a deep learning-enabled virtual histology image of prostate tissue. UV scattering and UV-PARS data channels for the area in the dashed box inset in (f) are shown in (g) and (i), respectively. Comparing the virtual histology inset in (h) to a true H&E-stained brightfield image in (j), we can again see that the stromal and nuclear details are concordant, with prostatic carcinoma clearly identifiable by pathologists. Virtual staining in both breast and prostate examples closely match the characteristic H&E coloration and also accurately emulate the translucent quality of brightfield microscopy, resulting in realistic images comparable to those which pathologists are accustomed and extensively-trained to interpret. Some differences between virtual and true histology images may be attributed to sample degradation following imaging as a result of tissue section dehydration/rehydration cycles and coverslip mounting, followed by coverslip removal and H&E staining for conventional H&E-stained brightfield image comparison.

**Fig. 4:**
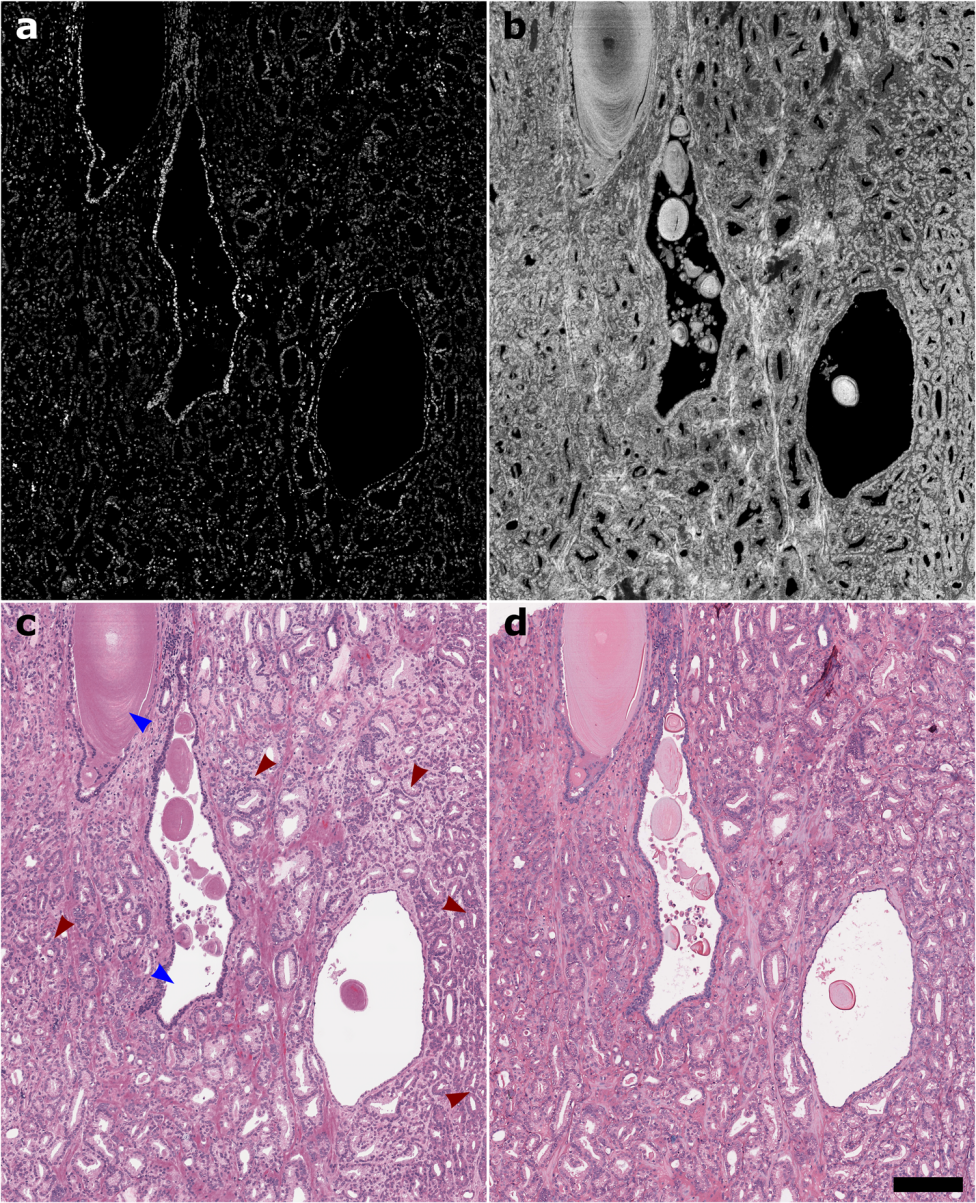
Fast large field-of-view imaging at sub-cellular resolution. **a** UV-PARS channel data. **b** UV scattering channel data. **c** Deep-learning enabled virtual histology image. **d** Corresponding true H&E-stained brightfield histology image. Pathologist annotations are included with red and blue arrows corresponding to prostatic carcinoma, and benign glands, respectively. Scale bar: 200 μm.

Figure 4c shows a large area unstained prostate deep learning-enabled virtual histology image with corresponding true H&E-stained brightfield histology image. These images are representative of images provided to pathologists for histological feature annotation and malignancy assessment. Figure 4a, b also show the UV-PARS and UV scattering channel data that were used as input to the trained CycleGAN network. Using our imaging approach we are able to acquire virtual histology at rates as fast as 7 mins/cm^2^, without sacrificing sub-cellular resolution. The image quality and the histological features of interest resolved in our images of unstained tissues were sufficient to identify regions of concern for malignancy, or favor a benign interpretation. Red arrows in 4c correspond to pathologist annotation indicating areas showing prostatic carcinoma, with blue arrows showing benign glandular features.

**Fig. 5:**
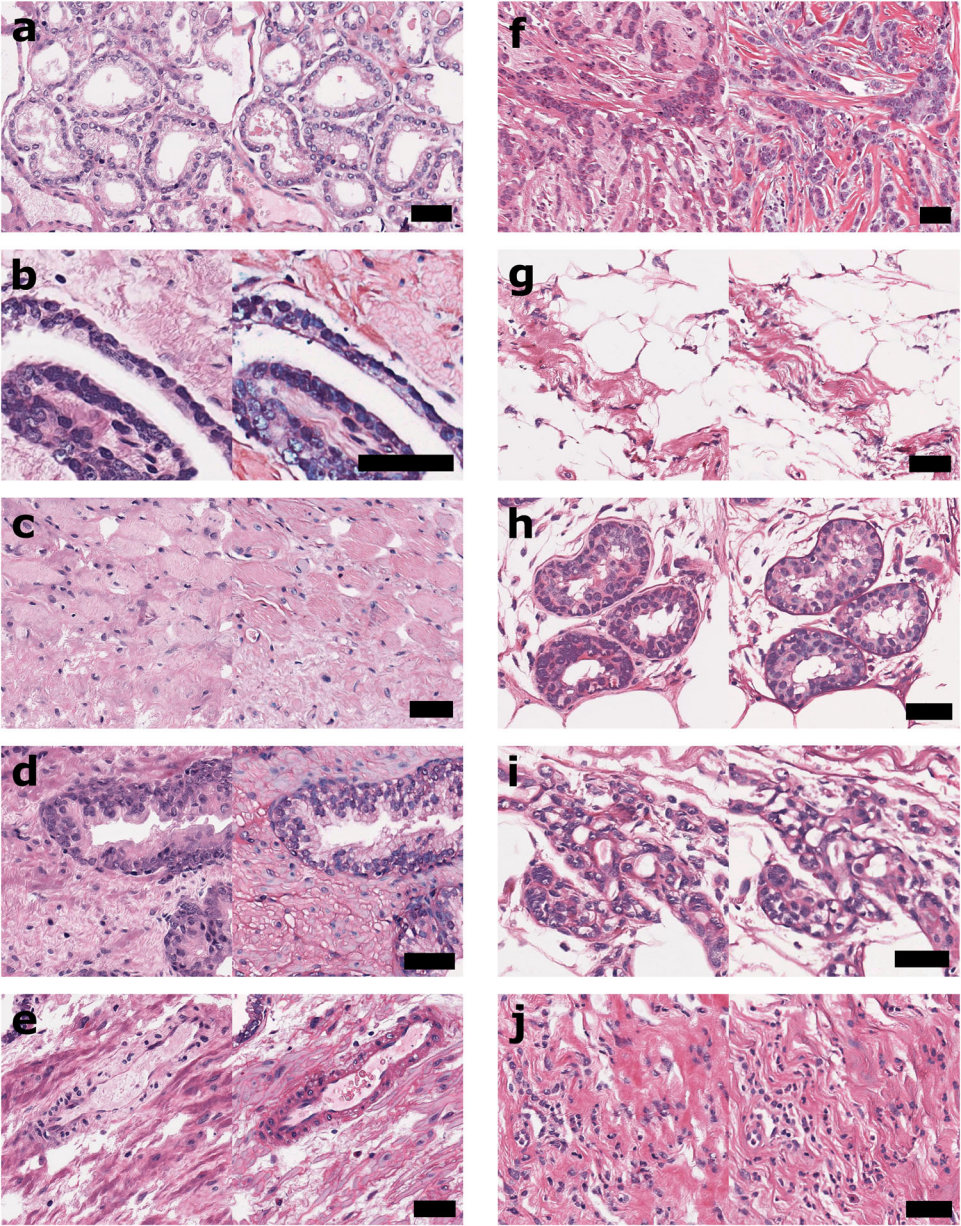
Concordant tissue features in virtual and gold standard histology. Representative examples of benign and malignant histological features of interest annotated by pathologists in both prostate (**a**–**e**), and breast (**f**–**j**) tissues, with virtual histology and corresponding true H&E-stained brightfield images shown on the left and right, respectively. Scale bars: 50 μm.

Pathologist annotation of our virtual images showcased the range of readily identifiable features which are important for diagnosis. Figure 5 displays a selected variety of these features appreciated in both benign and malignant prostate and breast tissues, with corresponding true H&E-stained brightfield comparisons. In these representative examples, pathologists were able to clearly identify in prostate tissues: (a) prostatic carcinoma, (b) perineural invasion, (c) benign stroma, (d) benign glands, and (e) benign blood vessels. Additionally, pathologists were able to clearly identify in breast tissues: (f) invasive ductal carcinoma, (g) fibroadipose tissue, (h) benign acini, (i) benign terminal duct lobular units, and (j) benign inflammation. Importantly, pathologists were able to discriminate benign and malignant cytologic features and evaluate tissue architecture in both tissue types.

**Fig. 6:**
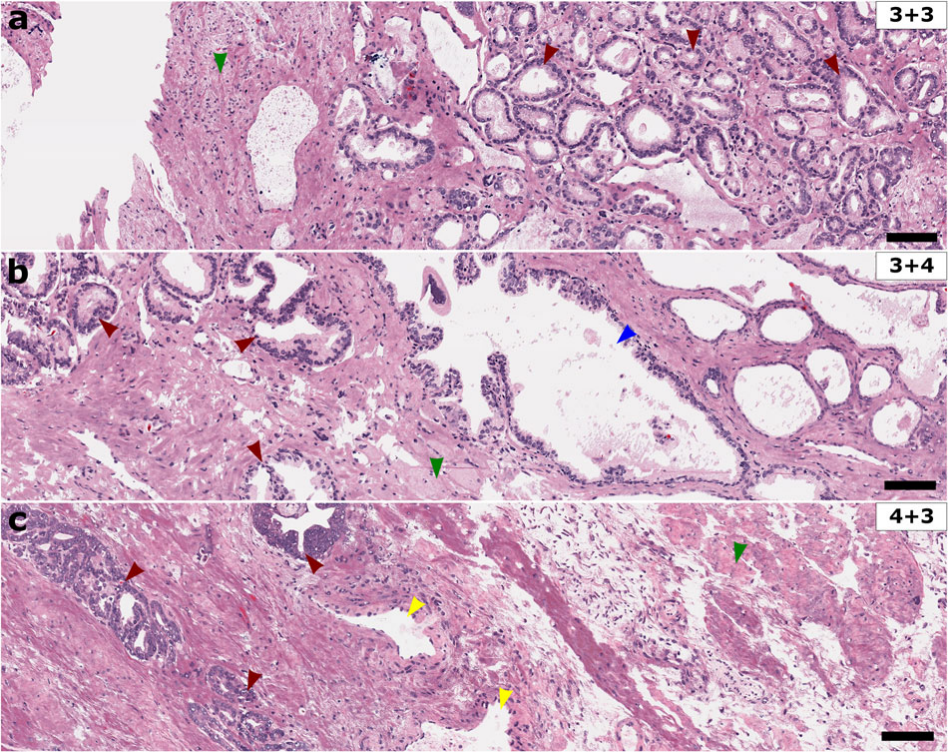
Gleason scoring in prostate virtual histology. Representative deep learning-enabled realistic virtual histology in unstained prostate tissues, with pathologist assigned Gleason scores of (**a**) 3+3, (**b**) 3+4, and (**c**) 4+3. Pathologist annotations are included with red, blue, green, and yellow arrows corresponding to prostatic carcinoma, benign glands, benign stroma, and benign blood vessels, respectively. Scale bars: 100 μm.

In addition, pathologists were able to assess Gleason scores for different virtual prostate tissue examples, with validation provided by matching assessments of the true H&E-stained brightfield counterparts. Figure 6 shows cropped examples of pathologist annotated samples with different assigned Gleason scores. Figure 6a shows a virtual histology image with corresponding Gleason score of 3+3, displaying regions of prostatic carcinoma (red arrows), and benign stroma (green arrow). Figure 6b shows a virtual histology image with assessed Gleason score of 3+4, displaying benign glandular features (blue arrow) and benign stroma (green arrow), as well as prostatic carcinoma (red arrows), with the presence of cribriform glands. Figure 6c shows a virtual histology image with corresponding Gleason score of 4+3, displaying cribriform glands (leftmost red arrows), and perineurual invasion (rightmost red arrow), as well as benign vasculature (yellow arrows), and benign stroma (green arrow).

### Virtual staining of freshly-resected thick murine tissues

**Fig. 7:**
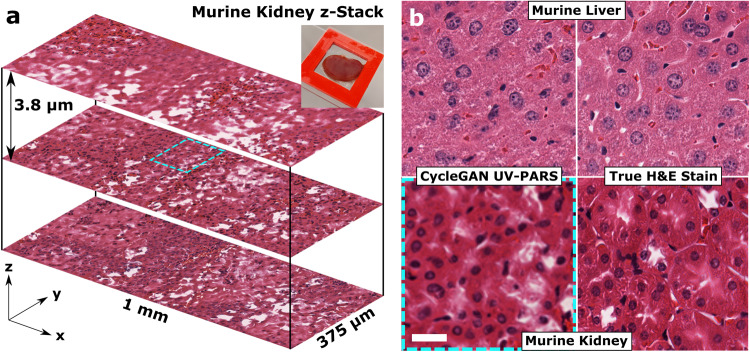
Multi-layer optical sectioning in mouse tissue virtual histology. **a** Z-stack of deep learning-enabled virtual histology images of freshly-resected thick murine kidney tissues, with a representative zoomed-in inset shown in (**b**) highlighted in the dashed box. Unpaired H&E-stained thin section brightfield images are shown for style comparison with virtual histology results in murine kidney and liver tissues. Scale bar: 25 μm.

In order to test the capability of this approach for imaging fresh, unprocessed thick tissues, a resected mouse kidney was imaged to generate a z-stack of en-face images in depth as shown in Fig. 7a. The resected tissue was placed between a microscope slide and UV-transparent coverslip as shown in the Fig. 7a inset, allowing us to rapidly flatten the tissue before imaging. This demonstrates the ability of the UV-PARS and UV scattering microscopy system to generate optically-sectioned multi-layer images. Note that direct morphological comparison to brightfield H&E histology is impractical given the difficulty in precisely maintaining tissue orientation through FFPE tissue processing, which is known to introduce changes in morphology. However, Fig. 7b shows a zoomed-in comparison of deep learning-enabled virtual H&E stains in both liver and kidney tissue images to comparable brightfield true H&E-stained thin tissue sections, illustrating the realism of the virtual stain even with images taken below the surface of thick, unsectioned tissues. Multi-layer UV-PARS imaging has been validated with co-scanned UV-PARS and confocal fluorescence microscopy^15^. Results additionally appear to support the success of the transfer learning approach in matching the virtual histological images to their true H&E-stained counterparts.

### Quantitative validation of virtual staining

**Fig. 8:**
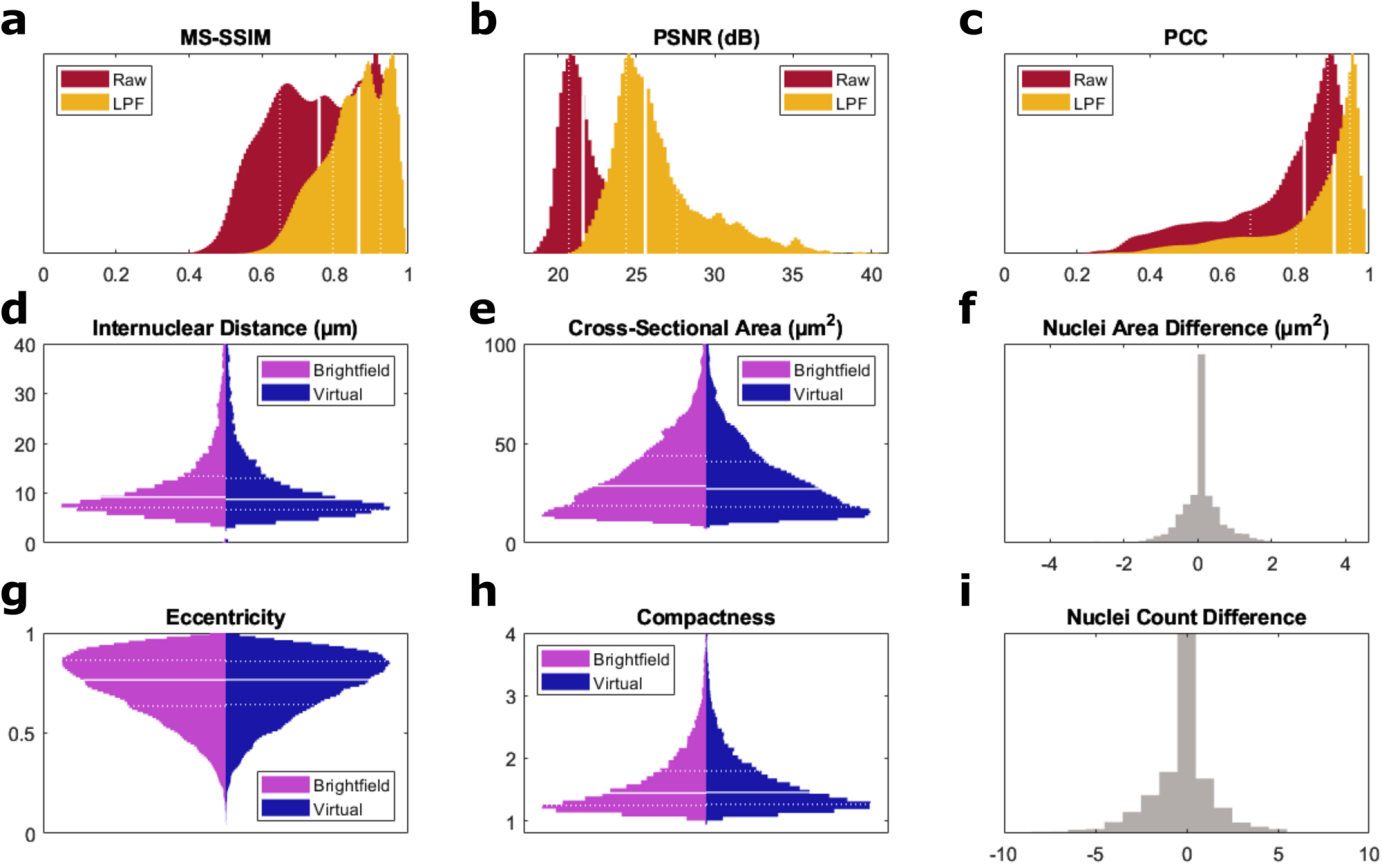
Quantitative validation of virtual histology. Metrics comparing 1921 pairs of ground truth brightfield H&E-stained and virtually-stained images, for raw images obtained at 390 nm resolution, and for low-pass filtered (LPF) images at an effective 2 μm resolution. **a** Normalized histogram of multi-scale structural similarity index measure (MS-SSIM). **b** Normalized histogram of peak signal-to-noise ratio (PSNR). **c** Normalized histogram of the Pearson correlation coefficient (PCC). Violin plots compare measured distributions for (**d**) internuclear distance, (**e**) cross-sectional nuclear area, (**g**) eccentricity, and (**h**) compactness. Normalized histograms show distributions of differences in (**f**) mean nuclear cross-sectional area and (**i**) nuclei count. Solid lines denote median values. Dashed lines denote 25^th^ and 75^th^ percentiles. Histogram bins are normalized as a probability density function. Source data are provided as a Source Data file.

To evaluate the performance of our deep learning-based virtual histology method, we quantitatively compared 1921 pairs of virtually-stained and corresponding H&E-stained images. For measuring perceptual similarity, the multi-scale structural similarity index measure (MS-SSIM) was computed for each image pair, in addition to the peak SNR (PSNR) and Pearson correlation coefficient (PCC). Example image pairs and associated quantitative metrics are shown in Supplementary Fig. 2. Histograms shown in Fig. 8a–c indicate our virtual histological staining offers similar visualizations to true brightfield H&E-stained histology, with a median MS-SSIM value of 0.76, a median PSNR of 21.6 dB, and a median PCC of 0.82 for raw images at 250 nm pixel spacing and full 390 nm optical resolution.

Additionally, we found that these metrics are improved at lower spatial resolutions. As such, we implemented low-pass filtering operations and re-computed the metrics at an effective 2 μm optical resolution to better compare our approach to alternative technologies (Supplementary Table 1) offering lower resolution. For these comparisons, we measured median values of 0.86, 26.5 dB, and 0.92, respectively, suggesting strong similarity of gross morphology despite slight contrast differences. Comparison of our quantitative metrics to alternative technologies is summarized in Supplementary Table 2, demonstrating favorable performance. It is important to note that there is some unavoidable loss in similarity that is unrelated to differences in the imaging modalities or due to sub-optimal CycleGAN performance. Such factors may arise from residual registration errors, degradation associated with multiple dehydration/rehydration cycles when imaging the unstained tissues with UV-PARS, and variability in the chemical staining process used to obtain the corresponding ground truth H&E-stained brightfield images. Additionally, the whole slide scans are not assured to be at an identical focus to the UV-PARS images, further contributing to possible discrepancies.

Nuclear morphology and spatial distribution metrics of diagnostic relevance were measured using the CellProfiler image analysis software^57^. The symmetry of the corresponding true and virtual H&E histograms shown as violin plots in Fig. 8 suggest a close match in the statistical distributions of cross-sectional nuclear area, morphological parameters including eccentricity and compactness, and the nearest neighbor internuclear distance. Further evidence of this is shown in histograms of the nuclear count and nuclear area differences between brightfield H&E images and our virtual histology in Fig. 8f, i. The majority of data is concentrated in the zero difference bins, and we find differences in nuclear cross-sectional area (<1 μm^2^) that are small relative to the median area (28.6 μm^2^). Some spread in these distributions is expected due morphological changes from the staining process and imperfect segmentation. Summary statistics and definitions of metrics used in this analysis are found in Supplementary Table 3. Additionally, we provide 2D spatial frequency domain comparisons between examples of corresponding true and virtual H&E histology images in Supplementary Fig. 3, where the virtual histology spatial frequency spectra generally match or offer enhanced bandwidth compared to the ground truth.

### Evaluation of diagnostic concordance

To evaluate the diagnostic concordance of our deep learning-enabled virtual histology method with gold standard H&E-stained histology, we provided pathologists with virtual histology images and their matching true H&E-stained counterparts for both prostate and breast tissues, with five and three pathologists providing feedback, respectively. Pathologists were asked to provide a malignant or benign diagnosis for each image in the data set. Pathologist consensus across true H&E histology images formed the gold standard diagnosis. Sensitivity, specificity, positive predictive value (PPV), negative predictive value (NPV), accuracy, and intra-observer concordance values were computed as outlined in Table 1. To reduce the effect of inter-observer variability, we report the mean value over all pathologists and additionally computed values using a consensus diagnosis for each virtual histology image. Referring to the breast tissue study in Table 1, our virtual histology method shows a high sensitivity of 0.96, arguably the most important metric for intraoperative pathology since it assesses the presence of false negatives, which are highly detrimental to patient outcomes in margin analysis. A high specificity of 0.91 suggests robustness of our method to false positives, which are also important to avoid in tissue-conserving procedures. A malignancy prevalence of 0.42 in the data set suggests we should obtain similar NPV and PPV to our calculated sensitivity and specificity, respectively, which is confirmed in the values obtained. Accuracy, an overall measure of diagnostic concordance including both false positives and false negatives, was measured to be 0.93, indicating low diagnostic error rates. Additionally, concordance values calculated using Cohen’s kappa suggest substantial intra-observer agreement between our virtual deep learning-enabled histology approach and the gold standard diagnosis^58^. In comparison to FSA in breast cancer cases, where a recent study^7^ determined a sensitivity of 0.78, specificity of 0.98, PPV of 0.65, NPV of 0.99, and accuracy of 0.97, our reported sensitivity and PPV were superior, and specificity, accuracy and NPV were comparable (>0.9). Some of these discrepancies may also be due to a lower malignancy prevalence in the frozen section study, which could result in inflating values such as the NPV. Detection performance in future work would also be expected to improve with pathologist access to larger areas of tissue in virtual histology images. The prostate tissue study in Table 1 shows high specificity and sensitivity values of 0.87 and 0.94, respectively, with a corresponding PPV of 0.97, and NPV of 0.82, and high accuracy of 0.90. Concordance values calculated using Cohen’s kappa again indicate substantial intra-observer agreement between our virtual deep learning-enabled histology approach and the gold standard diagnosis.

**Table 1:** Blinded pathologist reader study of diagnostic concordance

Breast tissue (*n* = 24 pairs)							
Malignancy prevalence = 0.42							
Ground Truth Fleiss’ Kappa^*^ = 1.00							
	P1	P2	P3	P4	P5	Mean	Consensus
Sensitivity	0.90	0.90	1.00	1.00	1.00	0.96	1.00
Specificity	0.93	0.86	1.00	0.86	0.93	0.91	0.93
Positive predictive value	0.90	0.82	1.00	0.83	0.91	0.89	0.91
Negative predictive value	0.93	0.92	1.00	1.00	1.00	0.97	1.00
Accuracy	0.92	0.88	1.00	0.92	0.96	0.93	0.96
Concordance (*κ*)^†^	0.83	0.75	1.00	0.83	0.92	0.86	–

Prostate tissue (*n* = 32 pairs)					
Malignancy prevalence = 0.63					
Ground Truth Fleiss’ Kappa^*^ = 0.81					
	P1	P2	P3	Mean	Consensus
Sensitivity	0.80	0.95	0.85	0.87	0.85
Specificity	1.00	0.83	1.00	0.94	1.00
Positive Predictive Value	1.00	0.90	1.00	0.97	1.00
Negative Predictive Value	0.75	0.91	0.80	0.82	0.80
Accuracy	0.88	0.91	0.91	0.90	0.91
Concordance (*κ*)^†^	0.75	0.80	0.81	0.79	–

Summary statistics from diagnostic concordance studies for breast and prostate tissue where a panel of pathologists (P) were tasked with interpreting paired virtual histology and ground truth H&E-stained brightfield images.

^†^Cohen’s kappa for intra-observer concordance.

^*^Fleiss’ kappa measures inter-observer concordance for interpreting ground truth H&E images. Source data are provided as a Source Data file.

### Blinded subjective survey of stain quality

**Fig. 9:**
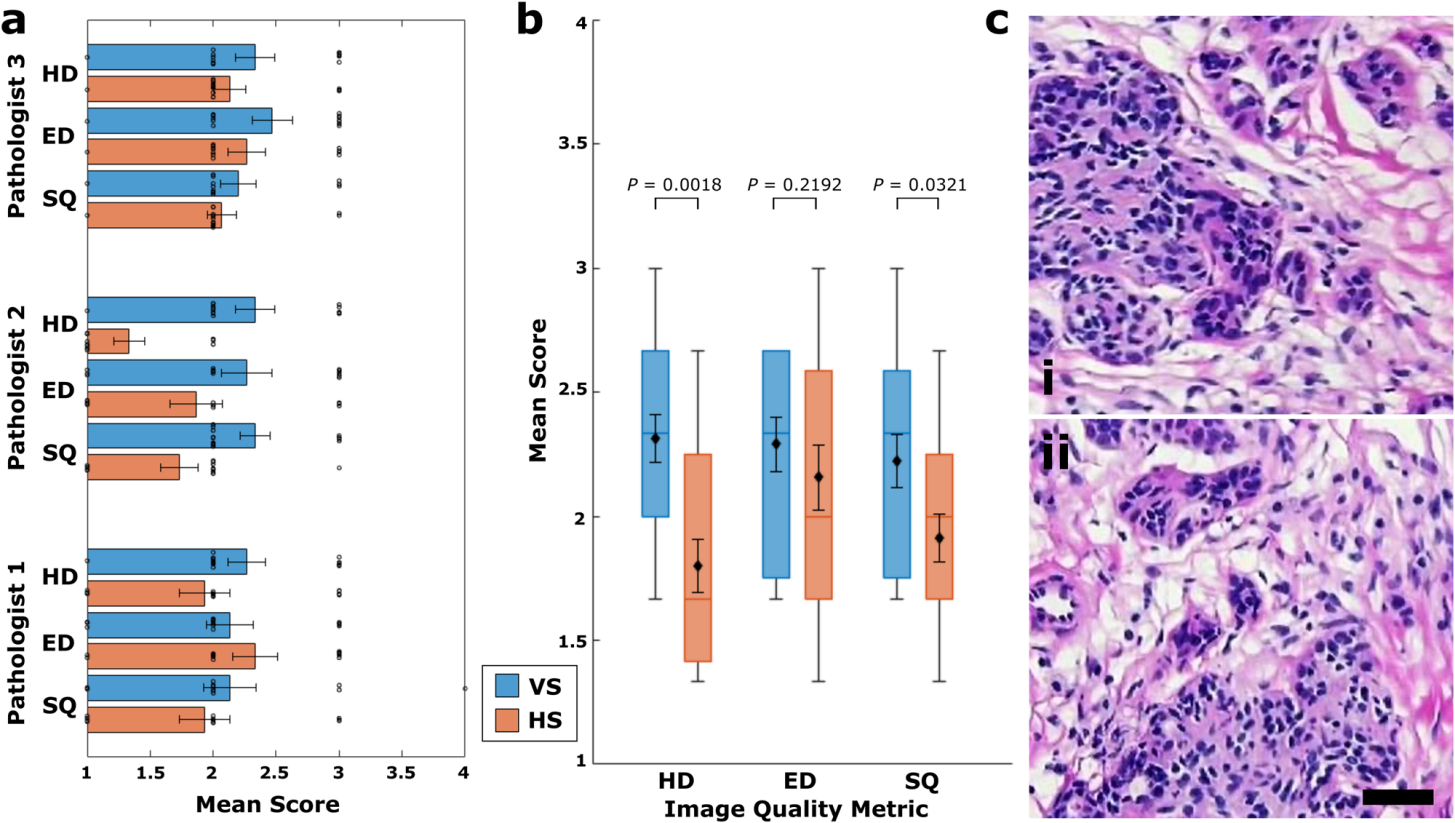
Blinded pathologist subjective stain quality survey. **a** Mean scores for each image quality metric provided by each pathologist for breast tissue virtual histology images of *n* = 15 independent specimens and *n* = 15 independent H&E-stained breast tissue frozen section specimens, with swarm plots showing the distribution of individual scores. Bars indicate mean ± SEM. **b** Box plots summarizing results for each histological imaging method and image quality metric using the inter-observer average image scores. Box center line and limits represent median and lower/upper quartiles, respectively. Whiskers represent lower/upper extrema, and no outliers are present. Diamond markers and associated errors bars report mean ± SEM. *P* values were determined using a right-tailed Wilcoxon rank sum test, without adjustments for multiple comparisons. SEM standard error of the mean, HD hematoxylin detail, ED eosin detail, SQ overall stain quality, HS histological stain, VS virtual stain. **c** Representative frozen section H&E-stained breast tissue brightfield microscopy. Scale bar: 50 μm. Source data are provided as a Source Data file.

To assess the subjective image quality of our approach, we compared deep learning-enabled virtual histology images to H&E-stained frozen sections, the current rapid intraoperative histology alternative in many potential use cases of our microscopy system. Representative examples of frozen section H&E-stained breast histology images are shown in Fig. 9c. A blinded pathologist study was conducted, where three pathologists were asked to rank image quality on metrics including hematoxylin detail, eosin detail, and overall stain quality on a scale of 1 to 4, with scores outlined in Supplementary Table 6. Figure 9a shows a summary of the mean scores provided by each pathologist for each of the image quality metrics. A sample size of *n* = 15 was used for each histological method. In comparing the mean scores between virtual and frozen section H&E-stained histology, the individual pathologists generally preferred the virtual stain approach in all but one result. It is worth noting that mean scores for virtual histology all exceed a threshold of 2 which was defined on the rating scale as acceptable stain quality, while the frozen section H&E-stained images failed to meet this standard. In addition, to mitigate the effect of inter-rater variability, an inter-pathologist average score was computed for each individual image and image quality metric. The resulting scores for all images in the *n* = 15 samples of each type are summarized in Fig. 9b, which suggests an overall preference for virtual histological imaging. Moreover, *P* values were determined using a right-tailed Wilcoxon rank sum test to compare the sample of images of each histological method across each image quality metric, with the null hypothesis taken as an equivalent or inferior scores for virtual staining compared to frozen sections. Hematoxylin detail and overall stain quality tests resulted in *P* values of 0.0018 and 0.0321, respectively, supporting the rejection of the null hypothesis at the 95% confidence level. This suggests a significant preference for virtual images with respect to these metrics. Although the mean eosin detail score for our virtual histology was higher than that for frozen section H&E-stained histology, the significance of this result was not established at the *α* = 0.05 level. Overall, the study results suggest that our deep learning-enabled virtual histology technique is preferred by pathologists over frozen section H&E-stained histology.

## Discussion

Our deep learning-enabled virtual histology approach offers promising results with multi-layered virtual histology capabilities and close concordance with true H&E-stained brightfield histology. The success of this method can be attributed substantially to achieving positive contrast to cell nuclei using UV-PARS, and high resolution in both contrast channels afforded by the 266 nm wavelength and laser scanning microscopy system architecture. The input images to the CycleGAN model already exhibit close concordance with true H&E-stained images, but deep learning helps achieve an enhanced level of realism which is validated using quantitative metrics and reader studies. As a future direction, providing class labels (e.g. benign vs. malignant, Gleason scores) or attention maps to the unpaired training data may also be explored for improving the GAN performance. Qualitatively, pathologists were able to readily identify important diagnostic features in virtual histology images, and assign Gleason scores to provided prostate samples. Additionally, diagnostic concordance pilot studies demonstrated strong performance in terms of sensitivity, specificity, PPV, NPV, accuracy, and intra-observer concordance for both breast and prostate tissues.

Results indicate that our method meets key functional requirements for microscopy systems proposed by the American College of Pathologists^59^, including resolution < 1 μm, a NPV of > 0.9 for margin assessment applications (breast tissue), and a PPV of > 0.9 for identifying lesional tissue for further studies or assessing core biopsy adequacy (prostate tissue). Furthermore, pathologists did not require additional training to interpret virtual histology presented in the familiar H&E-stained format, supporting the ease of use of this method.

As noted in the introduction, and summarized in Supplementary Table 1, our approach offers some advantages over other virtual histology systems. Methods which require staining or optical clearing procedures usually require an additional few minutes which can be eliminated in our label-free approach. Our rapid 7 mins/cm^2^ acquisition time is significantly faster than scan times reported for SRS microscopy^35,36^, recent UV-PAM imaging systems^43,46,48^, multispectral deep-UV microscopy^37^ or previous UV-PARS work^12–14,20^, while maintaining a high 390 nm resolution. Overall, our system offers a well-positioned trade-off of scan speed and resolution, while providing label-free contrasts that correspond well to gold-standard H&E-stained histology. We anticipate that higher resolution should also be possible without comprimising scan speed, using higher NA focusing and higher pulse repetition rate (PRR) lasers. This may enable our technology to better discriminate cytologic features.

Future embodiments will utilize parallelized scanning aiming to provide several-fold scan speed improvements, allowing higher throughput to move towards imaging multiple breadloafed surgical resection specimens within intraoperative time frames. Additionally, development of a cart-based system implementation will allow investigation of diagnostic utility in the clinical environment. Future work will also look to include additional contrasts of diagnostic interest beyond H&E histology. Collagen structure and NADH/FAD-linked metabolic indicators can be resolved through the detection of existing autofluorescence emission generated by our 266 nm excitation, and recent work suggests that biomechanical tissue properties can also be identified through elastography measurements from existing temporal UV-PARS signals^60^. This rich spectrum of information can be obtained simultaneously with a single scan of the sample.

Alternative approaches for intraoperative imaging include white-light surgical microscopy, fluorescence-guided surgery, and other virtual histology technologies. White-light surgical microscopy alone generally does not provide adequate tumor discrimination^61^. Surgical fluorescence imaging with dyes that are preferential to tumor tissues have shown promise, but sensitivity and specificity is still lacking, potentially as a result of biodistribution issues, background signal, and insufficient spatial resolution. For example, Ottolino-Perry et al. showed only 55.6% PPV for tumor identification, imaging a breadloafed lumpectomy specimen outside a demarcated tumor border where margin determination is critical^62^. In clinical pilot studies, we found sensitivity, specificity, and concordance were comparable to similar virtual histology modalities^26,35,63^, and greater than that reported for FSA as in Namdar et al.^7^.

Drawbacks of FSA have been well-documented. Frozen section artifacts often show atypical microscopic details that do not directly reflect an abnormality in the tissue and can make interpretation difficult^6^. For example, ice crystals can enlarge or introduce variability in nuclear size, and can cause hole formation mimicking the appearance of intracellular vacuoles or fat. Air drying artifacts can result in indistinct cell borders and smudged chromatin, while crush artifacts can also obscure histological details, hindering accurate evaluation. Moreover, shatter artifacts can occur if the cryostat temperature is not optimal or calcifications are present, where the tissue will appear similar to Venetian blinds. Adipose tissue in particular can be challenging to cut for FSA, often leading to tissue fragmentation and folding, or an increase in thickness. Given the technical limitations in quality and diagnostic utility, several studies have found false negative rates as high as 36% for frozen sections compared to permanent FFPE sections in certain procedures such as radical prostatectomy and breast-conserving surgery^3–5^. Poor agreement between frozen sections and permanent sections has also been noted in cancer types such as melanoma^64^. Our virtual histology with preferred stain quality offers promise as an alternative.

In summary, we developed a high-resolution, dual-contrast UV-PARS and UV scattering microscope which is able to produce virtual H&E-stained histology images with high quality and histological realism, aided by deep learning-enabled CycleGAN style transfer. A blinded pathologist study found that the nuclear detail and overall stain quality of our virtually-stained images were preferred over H&E-stained frozen sections with statistical significance (*α* = 0.05). Preliminary investigation of diagnostic concordance indicated our method offers high sensitivity, specificity, accuracy, and intra-observer concordance with conventional H&E-stained brightfield histology in both breast and prostate tissues.

Larger, well-powered clinical studies with a diversity of human subjects and clinical presentations are needed to rigorously establish diagnostic equivalence or non-inferiority of our virtual histology approach compared with frozen section and permanent section analysis. Unless non-inferiority of virtual histology methods can be demonstrated compared to conventional H&E-stained brightfield histology, virtual histology methods may not aim to supersede the gold standard as the primary method for definitive diagnosis, evaluating patient prognosis, and informing major clinical decision-making branch points such as treatment planning. However, virtual methods may excel for intraoperative margin analysis and situations where point-of-care analysis could be important for timely intervention or procedural guidance. In such applications, the current alternative is often only visual inspection or palpation, with negligible sensitivity to microscopic pathology. Here, virtual histology could address this unmet need for immediate microscopic analysis with improved false negative rates compared to gross evaluation. Moreover, our virtual histology is non-destructive unlike FSA, allowing initial interpretations to be confirmed with the confidence of gold standard histology once the time-intensive tissue preparation workflow can be completed.

Future work could also explore extending the image-to-image translation deep learning methods using a random noise seed or dropout-based stochasticity, to produce outputs that are increasingly non-deterministic^55^. This could offer utility that is synergistic with established brightfield histology practices, including the generation of high-quality teaching libraries for rare diagnostic entities that often contain only a few cases, and for generation of augmented data sets to extend and test extant machine learning pipelines that have been trained with real rather than synthetic morphological data.

Given the fast scan times, sub-cellular resolution, and pathologist-validated histological realism, our approach offers considerable promise for future intraoperative applications in margin assessment, especially as a surrogate for frozen sections.

## Methods

### Ethical statement

Human tissue specimens were obtained in accordance with approved ethics [HREBA (Cancer)/HREBA.CC- 20-0145], and murine tissues were obtained in accordance with protocols approved by the University of Alberta Animal Care and Use Committee [AUP00001170].

### Optical imaging system

A diagram of the optical setup used for simultaneous UV-PARS and UV scattering imaging is shown in Supplementary Fig. 1. In the second harmonic generation subsystem, a 10-2000 kHz PRR linearly-polarized 532 nm laser (SPFL-532-40, MKS) was focused through a 4 × 4 × 10 mm nonlinear cesium lithium borate (CLBO) crystal (Eksma Optics) to generate 266 nm excitation light. Residual 532 nm light was removed from the generated 266 nm beam via a dispersive prism (PS863, Thorlabs) and passed into a beam dump. The 266 nm beam width was then expanded using a Galilean beam expander to match the objective entrance pupil. A half-wave plate (WPH10M-266, Thorlabs) was used to rotate the polarization of the 266 nm beam to be maximally transmissive through a polarizing beam splitter cube (10SC16PC.22, Newport). The transmitted beam was then passed through a quarter-wave plate (WPQ10M-266, Thorlabs) converting linear into circular polarization. The circularly-polarized UV beam was then combined with an interrogation beam via a harmonic beam splitter (Di01-R355, Semrock), and both beams were co-focused onto the sample using a 0.50 NA reflective objective (LMM-40X-UVV, Thorlabs). Back-scattered circularly-polarized pulsed UV light was then redirected via the quarter-wave plate and polarizing beam splitter cube onto a photodiode (PDA10A, Thorlabs). By utilizing a custom sample-and-hold peak detector circuit, the photodiode measurement of the narrow UV pulse can be held and adequately sampled by the digital acquisition card. This back-scattered signal which utilizes the existing excitation wavelength for the UV-PARS subsystem is responsible for eosin-like contrast. A continuous wave (CW) 1060 nm center wavelength linearly-polarized superluminescent diode (SLD-1064-20-YY-350, Innolume) acted as the interrogation source, utilizing a circulator (HPBCIR-1060-H6-L-10-FA-SS, OF-Link) to redirect back-scattered light. By co-focusing both the interrogation and excitation beams, photoacoustic-induced reflectivity modulations can be detected using the AC-coupled output of a 75 MHz balanced photodiode (PDB420C-AC, Thorlabs), measuring back-scattered interrogation light intensity. This signal was further filtered using a in-line 22 MHz low-pass (BLP-21.4+, Mini-circuits), and 1.8 MHz high-pass filters (EF509, Thorlabs), where the latter filter was used to remove stage motion-induced reflectivity signals. This modulated CW interrogation back-scattered signal provides hematoxylin-like contrast. The axial and lateral resolutions of our system were measured as 1.6 μm^22^ and 390 nm^14^, respectively. Fast-axis voice coil stage (X-DMQ12L-AE55D12, Zaber) scanning was utilized in conjunction with slow-axis linear stage (XMS50-S, Newport) and its respective controller (XPS-DRV11, Newport) to enable rapid point-based scanning^21^. Z-axis translation was provided using a z-axis translation mount (SM1ZA, Thorlabs). Goniometers were utilized for both the fast (GN1, Thorlabs) and slow axis (#66-534, Edmund Optics) to align the stage translation axis to the sample surface.

### Data acquisition and processing

A function generator (DG1022Z, Rigol) and digital delay generator (DDG) (DG645, Stanford Research Systems) were used in combination to synchronize the excitation laser, digital acquisition (DAQ) card (CSE8389, Gage Applied), and peak detector reset. Channel 1 of the function generator was used to initiate 532 nm excitation lasing at a fixed PRR with a 50% duty cycle square wave, and as an external trigger to the DDG. The peak detector^65^ used to sample-and-hold back-scattered UV pulsed excitation light requires resetting between detection events to ensure no leakage of signal. This was achieved by providing a DDG time-delayed TTL signal, with a delay chosen outside the expected acquisition window. Channel 2 of the DDG was used to provide a time-delayed TTL signal to provide a reference to the DAQ of excitation events for post-acquisition image reconstruction. The DAQ utilized a 50 MS/s sampling rate to ensure the full PARS modulation and peak-detected UV back-scattered signals were captured. To properly resolve the voice coil stage AquadB encoder waveforms, data was acquired as a continuous stream rather than triggered. A custom multi-threaded C++ program implemented using the OpenMP API was developed for parallelized read-in of large raw data files, tracking stage position from encoder states, and associating the maxima of temporal data channel signals with a single spatial position to generate absorption and scattering data points on a sinusoidal scan trajectory. A parallelized MATLAB script was used to map this data onto a pixelated Cartesian grid using natural neighbor interpolation based on a Delaunay triangulation.

To generate input images for the CycleGAN, we first combine UV-PARS and UV scattering data into an RGB array, where UV-PARS data forms the red channel, UV scattering data forms the green channel, and the blue channel is not used. The complement of this RGB image is taken to match the brightfield background of true H&E histology and avoid the color inversion effect, a practical barrier found when training the CycleGAN^66^. An example of this input can be seen in Fig. 2a. In comparison to our previous approach of using a custom image fusion method^20^ based on a reference H&E-stained brightfield microscopy image, along with a blind stain separation algorithm^67,68^, this approach preserves orthogonality of the input data channels to avoid information loss. In practice, reasonable H&E images could be obtained by encoding the input UV-PARS and UV scattering data on any pair of the RGB channels for training the network, as the output color accuracy is enforced by the learned CycleGAN transformation. However, using the red and green channels appeared to optimize training results. We conjecture that this is due to color similarity to H&E stains following the image inversion step, minimizing the domain gap relative to true brightfield histology.

### Cycle-consistent generative adversarial network

The CycleGAN implementation used for translating UV-PARS images into realistic histological images was adopted from Zhu et al.^52^. As shown in Fig. 2b, the CycleGAN model consisted of the generators *G*_*UV*_ and *G*_*H&E*_, paired with discriminators *D*_*UV*_ and *D*_*H&E*_. The generators are trained to learn the translational mappings ${G}_{H{{{\&}}}E}:X\to \widehat{Y}$ and ${G}_{UV}:Y\to \widehat{X}$, where *X* is the domain of real UV-PARS images and *Y* is the domain of true H&E-stained brightfield histology images. Here, $\widehat{X}$ and $\widehat{Y}$ denote domains of translated output images, which ideally converge toward indistinguishable statistical distributions from the corresponding *X* and *Y* data domains under optimized generators. Adversarial training of each generator-discriminator pair was performed using least squares loss functions ${{{{{{{{\mathcal{L}}}}}}}}}_{adv}$^52^ on the discriminator outputs to compare candidate synthetic generator output images against input training image data (Fig. 2b).

The cycle consistency constraint necessary for unsupervised training on unpaired input data was enforced by cycle consistency loss functions ${{{{{{{{\mathcal{L}}}}}}}}}_{cyc}$^52^. These functions compute the mean absolute error of an input image *x* compared to its reconstruction *G*_*UV*_(*G*_*H&E*_(*x*)) following a round-trip cycle of the generative networks in the forward case, or similarly for input *y* with the reconstruction *G*_*H&E*_(*G*_*UV*_(*y*)) in the backward case. Additionally, an identity L1 loss term provided further regularization for matching the tint of the target domain^52^. The total loss function ${{{{{{{{\mathcal{L}}}}}}}}}_{tot}$ for the CycleGAN model used the following weighting for the individual terms:

1 $${{{{{{{{\mathcal{L}}}}}}}}}_{tot}=	{{{{{{{{\mathcal{L}}}}}}}}}_{adv}({G}_{UV},{D}_{UV})+{{{{{{{{\mathcal{L}}}}}}}}}_{adv}({G}_{H{{{\&}}}E},{D}_{H{{{\&}}}E})+\lambda {{{{{{{{\mathcal{L}}}}}}}}}_{cyc}({G}_{UV},{G}_{H{{{\&}}}E})\\ 	+0.5\lambda {{{{{{{{\mathcal{L}}}}}}}}}_{ident}({G}_{UV},{G}_{H{{{\&}}}E})$$

A heavy weighting hyperparameter for the cycle consistency loss, *λ* = 150, was tuned by experimental validation to ensure the tissue morphology of the input UV-PARS images is preserved under the translation, with the output primarily transforming the style into a realistic histological image.

The generator and discriminator network architectures of the CycleGAN are outlined in Fig. 2b. The generator networks are based on a ResNet architecture consisting of an encoder path for downsampling input images to an abstract latent space representation, nine residual blocks each with two convolutional layers for learning the transformation between image domains, and a decoder using fractionally-strided convolutions to re-construct output images. Each convolution used reflection padding and was followed by instance normalization and a ReLU activation, except the output where a hyperbolic tangent function was used to produce a [-1,1] bound. The discriminator networks used a 70 × 70 PatchGAN architecture. This network maps 256 × 256 input images to a 30 × 30 output array, where each element represents a classification that maps through the series of convolutions to overlapping 70 × 70 receptive fields in the input image. In this network, the first layer omitted the instance normalization, and leaky ReLU activation with a slope of 0.2 was used in all layers except the output. The output array was averaged to determine the classification value for the entire input image. The discriminator parameters were updated based on a stored buffer of 50 generated images rather than only the latest generated example, as suggested by Zhu et al. to reduce model oscillation^52^.

### Model training and inference

The training sets for UV-PARS virtual histology and true H&E-stained brightfield microscopy of breast and prostate tissues were generated by cropping 256 × 256 pixel patches from whole-slide digital pathology scans (Aperio, Leica Microsystems) using a sliding window. Scans were obtained at 400x magnification with an effective pixel resolution of ~ 247 nm. To increase diversity in the training sets, random transformations including rotations, reflections and positional jitter were applied to each patch. The breast tissue data set contained 16,000 unique images, and the prostate tissue data set included 20,000 unique images, sample sizes which exceed or compare to similar studies^34,38,46,48,49,56^. In each case, an 80:10:10 split was used for training, hyperparameter validation, and final testing, respectively using a random hold-out strategy. In constructing the data sets, we aimed for approximately equal proportions of benign tissues, and tissues obtained from tumor resection cases signed-out by a pathologist with a malignant diagnosis. This was done to ensure the network was presented with representative examples of both benign and malignant histology, mitigating potential bias in the style transfer output, and potentially reducing the occurrence of hallucinations known to be associated with over or under-represented classes in the training data^69^. While the model was trained on small high-resolution patches, the fully-convolutional nature of the network permits style transfer of arbitrarily large images at the inference stage, limited only by GPU memory.

For training the mouse tissue style transfer models for thick tissue imaging, 512 × 512 pixel grids over a 125 μm × 125 μm field of view were used for each image. The liver tissue training set consisted of 200 each of UV-PARS virtual histology images and true H&E-stained brightfield images. The kidney tissue training set contained 300 images of each type. Given the reduced amount of training examples in these cases, a transfer learning approach was employed. While the human breast and prostate translation models were trained from scratch, the mouse tissue translation models used the fully-trained human breast tissue model as a starting point. While these images differ considerably in style, they contain similar morphological features, which we expect to aid the progression of the model training. Due to the fully-convolutional nature of the networks used, the number of learnable parameters is insensitive to the spatial dimensions of the input data. Thus, the pre-trained breast tissue model could be updated using larger 512 × 512 mouse tissue training patches, which empirically produced the best visual results.

In each case, the CycleGAN model was trained to convergence using the Adam optimization algorithm with a batch size of 1, and moment parameters of 0.5 and 0.999. Training persisted for 100 epochs at a learning rate of 0.0002, with learning rate decay to zero for a further 100 epochs using a linear policy. Training the breast and prostate tissue translation models from scratch required ~ 57 h using Nvidia Tesla A100 GPU resources from Google Colab Pro+. Following training, only the optimized *G*_*H&E*_ generative model is needed for subsequent style transfer of new UV-PARS images to realistic virtual histology, requiring only up to a few seconds. For style transfer in high-resolution, large field-of-view images exceeding GPU VRAM constraints, images were divided into horizontal rectangular strips with 256 overlapping pixels, then re-stitched using linear blending following the CycleGAN transformation to produce a seamless output image. This approach facilitated by the fully-convolutional network architecture is more efficient than splitting the image into small squares, as it uses the maximal area for each patch within memory constraints, and reduces the total area of overlapping boundaries, eliminating vertical borders entirely. These factors serve to mitigate potential image degrading effects including stitching artifacts, residual visible seams, and brightness differences between patches.

### Tissue acquisition and preparation

Formalin-fixed, breadloafed human breast tumor lumpectomy and radical prostatectomy tissue specimens were obtained from breast and prostate cancer patients, after pathology cases were closed and tissues were otherwise flagged for disposal as per approved ethics [HREBA (Cancer)/HREBA.CC- 20-0145]. Breast tissue specimens represented 9 female subjects ranging in age from 35 to 69. Prostate tissue specimens included cases from 7 male patients ranging in age from 55 to 71. Samples representative of benign breast histology were obtained from a reduction mammoplasty procedure, where the tissue would have otherwise been discarded. Prostate tissues were obtained exclusively from males who have had a radical prostatectomy procedure. Breast tissues were obtained exclusively from female subjects due to the relatively lower incidence of breast cancer in the male population. This study did not include direct human research participants, but rather involved secondary analysis of anonymized tissue samples and pathology reports. Accordingly, informed consent was not required by the Health Research Ethics Board of Alberta (HREBA) in the approved research ethics certification. Tissue specimens were paraffin-embedded and sectioned into 4 μm thin sections, which were H&E-stained for morphological comparison where possible, following label-free imaging. De-paraffination of tissue sections was carried out by heat-adhering the tissues to the slides at 60 ^∘^C for 1 h, followed by 5 min washes in two changes of xylene, two changes of 100% ethanol, 95% ethanol, and deionized water. Deparaffinated sections were then re-hydrated prior to imaging. Murine tissues were obtained in accordance with protocols approved by the University of Alberta Animal Care and Use Committee [AUP00001170]. Fresh murine kidney and liver specimens were immersed in a phosphate-buffered saline solution immediately after dissection from a Swiss Webster mouse (Crl:CFW(SW), Charles River Laboratories) following isofluorane-induced euthanasia. Freshly-resected tissues were imaged using UV-PARS within 30 min, prior to formalin fixation.

### Analysis of image similarity and nuclear morphology metrics

A set of *n* = 1921 paired image patches was used to compare our virtual histological staining approach with conventional H&E-stained brightfield histology. Images were initially coarsely aligned by maximizing the normalized cross-correlation. Image co-registration was further refined using an evolutionary algorithm for 1000 iterations to optimize intensity-based Mattes’ mutual information metric^70^ under an affine transformation with 64 padding pixels used to account for registration degrees of freedom before cropping to 256 × 256 pixels. Finally, non-rigid pixel-level correction was applied using cubic interpolation and a displacement field estimated by Thirion’s demons algorithm^71^ with 100 iterations, 3 multi-resolution pyramid levels, and an accumulated field smoothing parameter of 2.0. Finally, a manual data cleaning step was performed to remove anomalies such as tissue tearing, the presence of red blood cells or dust particles, and defocusing during whole slide scanning. The MS-SSIM, PSNR, and PCC were computed to quantify the similarity of each image pair. PSNR was calculated for RGB images, while the PCC was determined using the grayscale image luminance. For the MS-SSIM calculation, images were converted to the *YC*_*b*_*C*_*r*_ color space, then the channel-wise results were combined as $0.8SSI{M}_{Y}+0.1SSI{M}_{{C}_{b}}+0.1SSI{M}_{{C}_{r}}$ following the weighting convention suggested for color images by Wang et al.^72^. This analysis was performed for raw images at full 250 nm sampling resolution, and additionally for Gaussian filtered images simulating a reduced optical resolution of 2 μm to characterize gross morphological similarity, while discounting the effects of contrast and focusing differences between the modalities.

For characterizing diagnostically-relevant metrics describing the morphology and distribution of cell nuclei in our virtual histological images, as compared to ground truth brightfield H&E-stained images, an analysis pipeline was developed using the CellProfiler software. A stain separation algorithm^67,68^ was used prior to CellProfiler processing, retaining only the hematoxylin stain to assist the segmentation of nuclei. Initially, the complement of the grayscale images was taken such that nuclei appear brighter against a darker background. The IdentifyPrimaryObjects function was used for detection of nuclei, with a typical object diameter range of 15–50 pixels. Global thresholding by Otsu’s method was used with a smoothing scale parameter of 1.35, a correction factor of 0.8, and intensity threshold bounds of 0.1 to 1.0. Clumped objects were distinguished and dividing lines were drawn using the shape method^57^. A nuclei count was determined by the total number of detected objects, while discarding objects outside the specified size range or in contact with the image border, which could limit accurate morphological measurements. The MeasureObjectSizeShape module was used to measure the cross-sectional nuclear area using the set of detected objects. Measures of nuclear shape including eccentricity and compactness were also computed, with definitions provided in Supplementary Table 3. Finally, the MeasureObjectNeighbors module was used to characterize the internuclear distance using the nearest neighbor for each nucleus object centroid. All parameters were computed over the distribution of 1921 images of each type, with results shown in Fig. 8.

### Evaluation of diagnostic concordance

To evaluate the diagnostic utility of our virtual histology images in breast tissues, we designed a study where five pathologists were asked to provide a malignant or benign diagnosis in *n* = 24 virtual H&E images with matching true H&E counterparts. Results are summarized in Supplementary Table 4. For prostate tissues, we designed a study where three pathologists were asked to provide a malignant or benign diagnosis in *n* = 32 virtual H&E images with matching true H&E counterparts. Results are summarized in Supplementary Table 5. Images selected for these studies were obtained by imaging random regions of tissue sections unseen by the CycleGAN model during training, while ensuring data sets represented a diversity of histological features. The field-of-view presented to pathologists for paired virtual and true H&E histology ranged from 1 to 10.7 mm^2^, with a mean of 3.7 mm^2^. In both studies, images were from benign and malignant tissues as validated by blinded pathology reports. In order to establish a benchmark diagnosis, the consensus interpretation was selected across pathologists for the true H&E image set. Pathologists had good agreement in interpreting true H&E images, as quantified by a calculated Fleiss’ kappa score of 1.00 for breast tissue and 0.81 for prostate tissue. This supports using the consensus diagnosis in the true H&E images as a gold standard. Furthermore, the gold standard diagnoses indicated a malignancy prevalence of 0.42 in the breast data set and 0.63 in the prostate data set, suggesting good representation of both malignant and benign samples. We evaluated the diagnostic utility of our virtual histology by calculating the sensitivity, specificity, PPV, NPV, and accuracy for interpretations by each pathologist. Additionally we determined the concordance of pathologist interpretations of true histology with their own interpretations of corresponding virtual histology using Cohen’s kappa as a measure of intra-observer variability. To mitigate inter-observer variability, we report means of these values over all pathologists, and additionally computed these statistics using a consensus diagnosis for each virtual histology image. Results are shown in Table 1.

### Blinded comparison of virtual histology and frozen section stain quality

A set of *n* = 15 frozen section H&E-stained brightfield microscopy images of human lumpectomy specimens obtained from a tissue bank (OriGene), and *n* = 15 CycleGAN UV-PARS virtually-stained images of human breast specimens obtained from benign tissues and tumor lumpectomy procedures were compiled to form a randomized blinded set of breast histology images. These images were then presented to three pathologists who were asked to rank each image based on hematoxylin detail (HD), eosin detail (ED), and overall stain quality (SQ) on a 1–4 evaluation scale, similar to the study reported by Rivenson et al.^34^. The numerical scale defined scores as follows: 1, unacceptable; 2, acceptable; 3, very good quality; and 4, perfect stain. The results of this blinded evaluation of virtual and frozen H&E-stained breast tissue images are tabulated in Supplementary Table 6. To evaluate prospective statistical significance of the hypothesis that virtual images produced image quality that was preferred over frozen sections by pathologists, a right-tailed Wilcoxon rank sum test was performed, which is generally considered as the nonparametric version of a two-sample *t* test. This test is appropriate for comparing independent samples that may not be normally-distributed, allowing the significance of test statistic differences in populations to be evaluated where the distributions have approximately equal variances. This assumption appears to be reasonable in our study when sample variances are used as an estimate of population variances. A test was performed for each stain quality metric, using the exact version of the test statistic computation. Moreover, tested image scores were taken as a mean over pathologist ratings, which in part reduces the effect of inter-observer variability, and avoids the problem of computing *P* values where ties exist in the exact Wilcoxon rank sum test. The experimental design involved only three planned comparisons, therefore no corrections for multiple comparisons were used.

### Statistics & reproducibility

All images depict single scans of single samples to demonstrate proof of concept, though all data sets are representative of multiple repeated experiments where the same histological features were consistently resolved. In all studies, pathologist readers were blinded to the imaging method and final diagnosis for the cases from which specimens were obtained. No statistical method was used to predetermine sample sizes. Image data was excluded only in cases where the 400x magnification whole-slide scan was out of focus, dust particles were present in the field of view, or the image patch contained only glass slide/coverslip and not tissue.

### Reporting summary

Further information on research design is available in the Nature Portfolio Reporting Summary linked to this article.

### Supplementary information

Supplementary Information

Reporting Summary

### Source data

Source Data

## Data Availability

Source data are provided with this paper as a Source Data file. The training, test, and validation data sets for the breast tissue and prostate tissue staining models are available at 10.5281/zenodo.7981075. Due to their large file size, the raw data generated during this study are available for research purposes from the corresponding authors on request, within three weeks. Source data are provided with this paper.

## Code availability

The code for CycleGAN originally implemented by Zhu et al. in PyTorch^52,55^ is available at https://github.com/junyanz/pytorch-CycleGAN-and-pix2pix. This model was trained for our application with customized parameters as described in Methods. The pre-trained breast and prostate CycleGAN virtual-to-true H&E generator networks are available at 10.5281/zenodo.8115040. The data processing and analysis described in Methods were implemented in MATLAB scripts which are available from the authors upon request. Quantitative analysis of nuclei was performed using the free, open-source CellProfiler software. The system control and image reconstruction software are available from the corresponding author upon request. The requester is responsible for procuring all necessary licenses for third party libraries and software development kits.
