# Supplementary material for: Deep learning-enabled realistic virtual histology with ultraviolet photoacoustic remote sensing microscopy
Source: Nat Commun. 2023 Sep 25;14:5967. doi: 10.1038/s41467-023-41574-2 (PMC10519961; doi:10.1038/s41467-023-41574-2)
Supplement: Supplementary file 1 — Supplementary Information [file 41467_2023_41574_MOESM1_ESM.pdf]

Contents

List of Figures

1 UV-PARS and UV scattering combined system architecture . . . 2
2 Example quantitative comparison of paired virtual and true H&E-stained histology images . . . 3
3 Frequency domain comparison for representative examples of virtual and true H&E-stained histology . . . 4

List of Tables

1 Comparison of key technical specifications for current virtual histology platforms . . . 5
2 Comparison of reported quantitative metrics for alternative virtual staining techniques . . . 6
3 Quantitative nuclei metrics for brightfield H&E and virtual histology comparisons . . . 7
4 Deep learning-enabled breast tissue virtual histology diagnostic concordance study results . . . 8
5 Deep learning-enabled prostate tissue virtual histology diagnostic concordance study results . . . 9
6 Blinded pathologist survey of subjective stain quality . . . 10

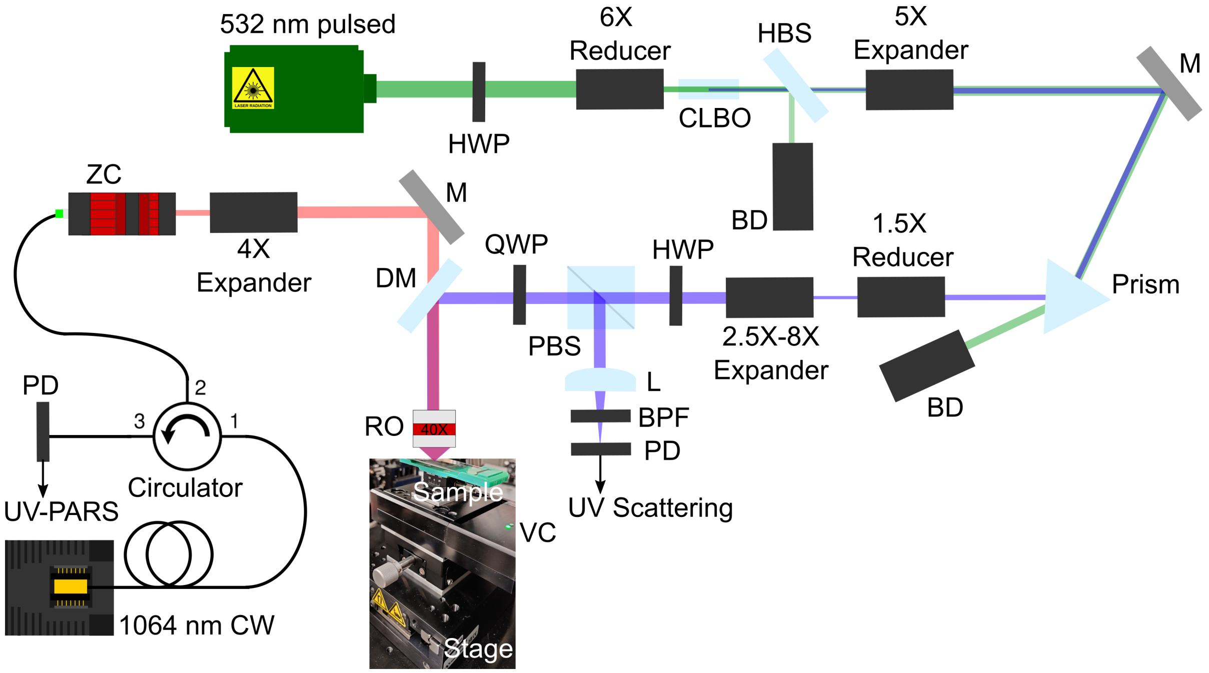

Figure 1. UV-PARS and UV scattering combined system architecture. L, lens; HBS, harmonic beam splitter; CLBO, cesium lithium borate crystal; M, mirror; HWP, half-wave plate; QWP, quarter-wave plate; BPF, bandpass filter; PBS, polarizing beam splitter; PD, photodiode; RO, reflective objective; ZC, zoom collimator; BD, beam dump; VC, voice coil stage; DM, dichroic mirror.

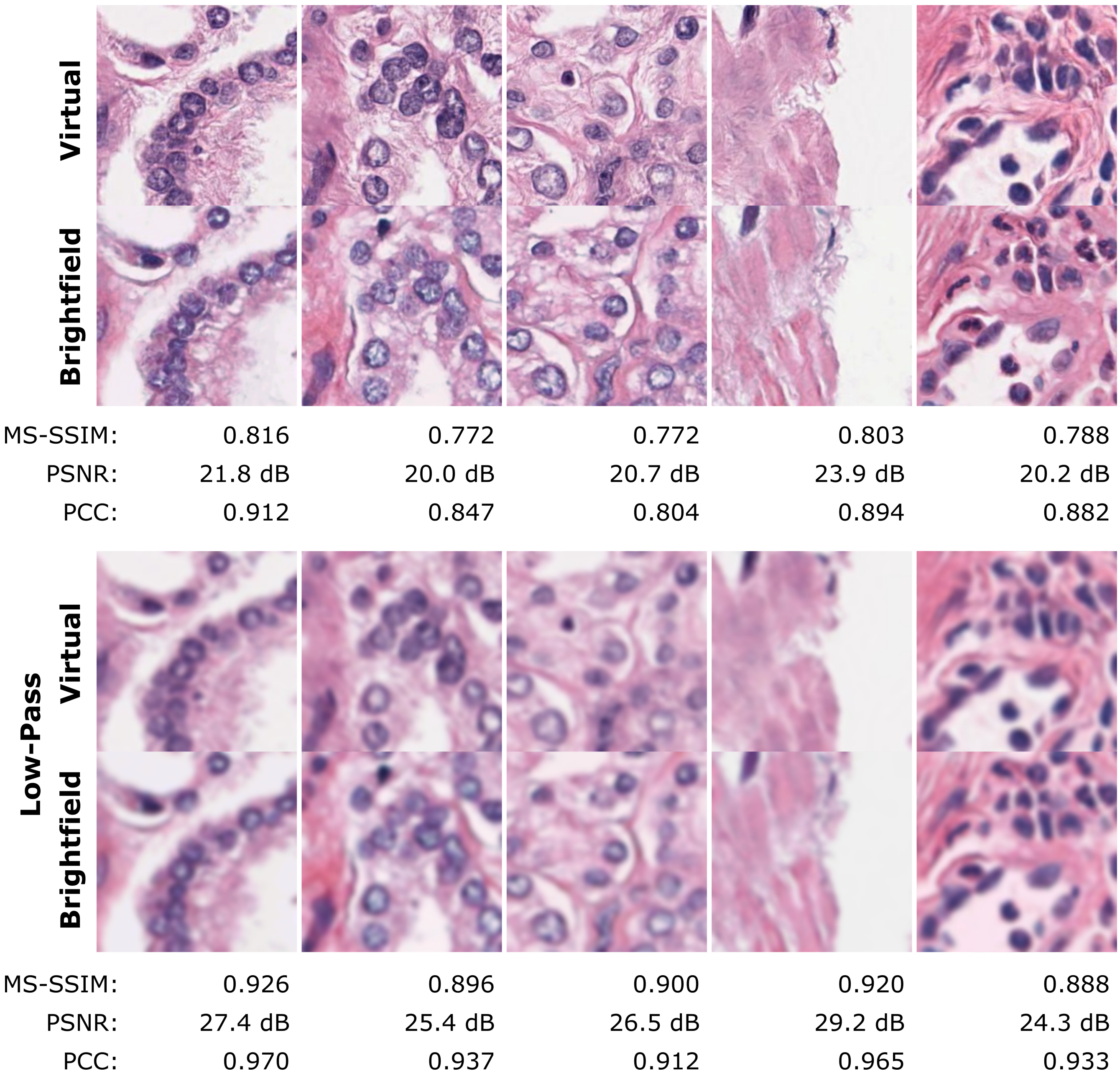

Figure 2. Example quantitative comparison of paired virtual and true H&E-stained histology images. Measured results for raw images at 390 nm optical resolution and a 250 nm pixel spacing are shown (top), in addition to low-pass Gaussian filtered results simulating an effective 2 μm optical resolution (bottom). MS-SSIM, multi-scale structural similarity index measure; PSNR, peak signal-to-noise-ratio; PCC, Pearson correlation coefficient.

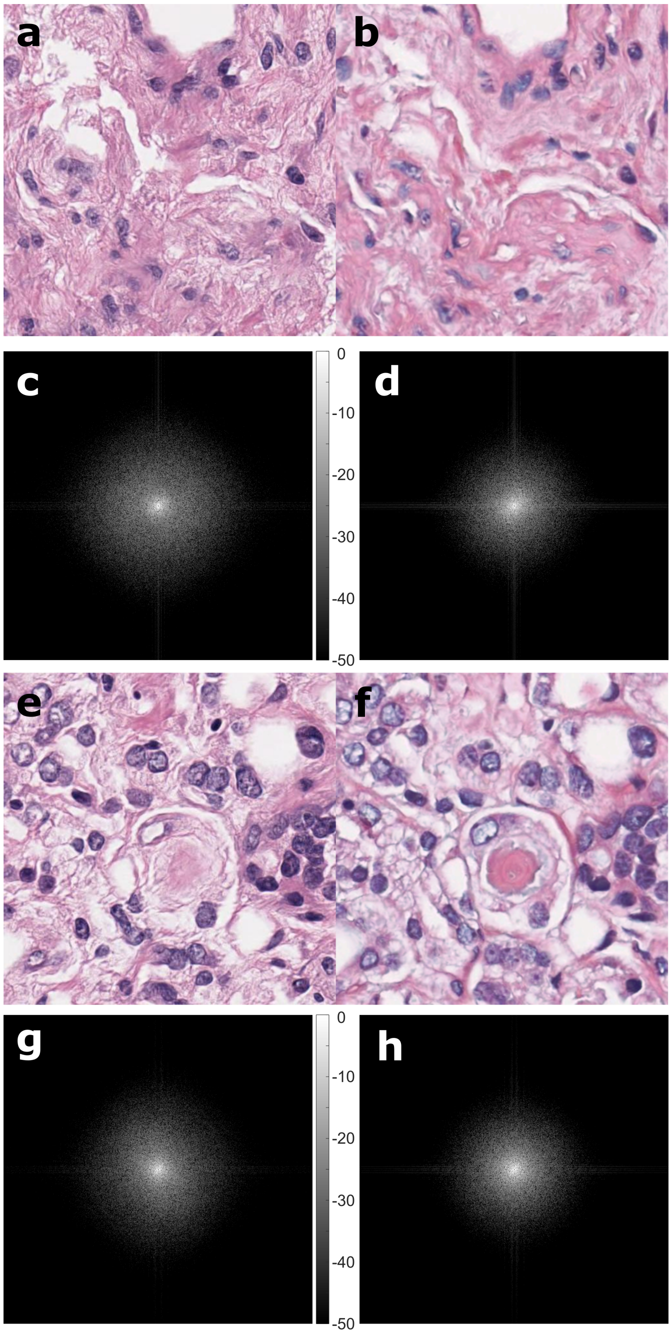

Figure 3. Frequency domain comparison for representative examples of virtual and true H&E-stained histology. a) Virtual histology image. b) True H&E-stained histology image corresponding to a). c-d) Log-scale 2D spatial frequency spectrum of a) and b), respectively. e) Virtual histology image. f) True H&E-stained histology image corresponding to e). g-h) Log-scale 2D spatial frequency spectrum of e) and f), respectively.

Imaging Modality	Mechanism	Scan Time per cm^2	Lateral Resolution	Label-Free	Multiple Contrast Channels	Diagnostic Validation	References
**UV-PARS**	Non-Radiative Absorption + Elastic Scattering	7 mins	390 nm	✓	✓	✓	This Work
**Open-Top Light-Sheet Microscopy**	Fluorescence	12.5 s	1.5-1.8 μm	×	✓	✓	1,2
**Confocal Microscopy**	Fluorescence/ Scattering	45 s†/ 6.25 mins	1.25 μm/2 μm	✓/×	✓/×	✓	3–5
**MUSE**	Fluorescence	53-80 s	600 nm	×	✓	×	6
**SIM**	(Auto)fluorescence	14 s†/ 1.7 mins†	1.3/1.1 μm	✓/×	×	✓	7,8
**OCT/OCM**	Elastic Scattering	45 s / 5.2 mins†	20 μm/2 μm	✓	×	✓	9–11
**MediSCAPE**	(Auto)fluorescence	2-3 mins	0.8-1.1 μm	✓/×	✓	×	12
**NLM/SLAM**	Multiphoton Fluorescence/ Multiharmonic Generation	8.9 mins/ 27.2 mins†	440/500 nm	✓/×	✓	✓	13–15
**UV-PAM**	Non-Radiative Absorption	14.2 mins†- 16.7 hrs	330 nm**- 1.6 μm	✓	×	×	16–19
**Multispectral Deep-UV Microscopy**	Absorption + Elastic Scattering	17 mins†	300 nm	✓	✓	✓	20
**SRS**	Inelastic Scattering	3.3 hrs	360 nm	✓	✓	✓	21,22
**TA-PARS**	Absorption + Elastic Scattering	11.1 hrs	350 nm	✓	✓	✓	23–27

Table 1. Comparison of key technical specifications for current virtual histology platforms. It is important to note that specifications represent implemented trade-offs in real reported system designs, but not necessarily fundamental limitations. †Lower-bound on effective cm^2 scan times extended from reported field-of-view imaging times: does not account for translational motion between tiles for image mosaicking, or overlap required for image stitching. **Transmission-mode only.

Modality	(MS-)SSIM	PSNR	PCC	Reference
**UV-PARS (390 nm Resolution)**	0.76	21.6 dB	0.82	This Work
**UV-PARS (2 μm Resolution)**	0.86	25.6 dB	0.90	This Work
**Autofluorescence**	0.9	-	-	28
**Autofluorescence**	0.38-0.57	13.6-21.7 dB	-	29
**TPEF + FLIM**	0.71-0.76	-	-	30
**CARS + TPEF + SHG**	0.6	-	-	31
**RCM**	0.55	-	0.56	5
**Brightfield**	0.58	21.5 dB	-	32
**QPM**	0.81-0.89	-	-	33
**QPM**	0.853*	-	-	34
**Deep-UV Microscopy**	0.91*	-	-	35
**TA-PARS**	0.91†	-	-	24
**MUSE**	0.27-0.37	19.0-19.5 dB	0.71-0.73	36
**CHAMP**	-	-	0.7-0.9	8

Table 2. Comparison of reported quantitative metrics for alternative virtual staining techniques. *Imaging of single cells or smears rather than tissues may inflate results where uniform background forms large fraction of images. †Analysis performed in L^*a^*b colorspace which may bias values.

Metric	Definition	Brightfield H&E	Virtual H&E
**Cross-Sectional Area**	A	28.6 μm^2	27.1 μm^2
**Eccentricity**	$\sqrt{1-\frac{b^2}{a^2}}$	0.765	0.766
**Compactness**	$\frac{\overline{D(p_i,c)^2}}{A}$	1.44	1.45
**Internuclear Distance**	$D(c_i,c_j)$ where i,j are nearest neighbors	9.2 μm	8.7 μm

Table 3. Quantitative nuclei metrics for brightfield H&E and virtual histology comparisons. Median values of nuclear size and shape metrics for true brightfield H&E histology as compared to our virtual histology method. a, length of semi-major axis; b, length of semi-minor axis (for ellipses with identical second central moments to segmented region); A, area. $D(x,y)$, Euclidean distance function; c, position of segmented object centroid; p_i, position of ith pixel. Overbar denotes mean taken over segmented object pixels.

	True H&E-stained histology						Virtual histology					
Image	P1	P2	P3	P4	P5	Consensus	P1	P2	P3	P4	P5	Consensus
1	B	B	B	B	B	**B**	B	B	B	B	B	**B**
2	M	M	M	M	M	**M**	M	M	M	M	M	**M**
3	B	B	B	B	B	**B**	B	B	B	B	B	**B**
4	M	M	M	M	M	**M**	B	B	M	M	M	**M**
5	M	M	M	M	M	**M**	M	M	M	M	M	**M**
6	B	B	B	B	B	**B**	B	B	B	B	B	**B**
7	M	M	M	M	M	**M**	M	M	M	M	M	**M**
8	B	B	B	B	B	**B**	B	B	B	B	B	**B**
9	M	M	M	M	M	**M**	M	M	M	M	M	**M**
10	B	B	B	B	B	**B**	B	B	B	B	B	**B**
11	M	M	M	M	M	**M**	M	M	M	M	M	**M**
12	B	B	B	B	B	**B**	B	B	B	B	B	**B**
13	B	B	B	B	B	**B**	B	B	B	B	B	**B**
14	B	B	B	B	B	**B**	B	B	B	B	B	**B**
15	M	M	M	M	M	**M**	M	M	M	M	M	**M**
16	B	B	B	B	B	**B**	B	M	B	B	B	**B**
17	M	M	M	M	M	**M**	M	M	M	M	M	**M**
18	M	M	M	M	M	**M**	M	M	M	M	M	**M**
19	M	M	M	M	M	**M**	M	M	M	M	M	**M**
20	B	B	B	B	B	**B**	B	B	B	B	B	**B**
21	B	B	B	B	B	**B**	M	M	B	M	M	**M**
22	B	B	B	B	B	**B**	B	B	B	B	B	**B**
23	B	B	B	B	B	**B**	B	B	B	B	B	**B**
24	B	B	B	B	B	**B**	B	B	B	M	B	**B**

Table 4. Deep learning-enabled breast tissue virtual histology diagnostic concordance study results. Pathologist diagnostic concordance study results for deep learning-enabled breast tissue virtual histology images and true-H&E stained counterparts, with a consensus value representing the mode of pathologist (P) interpretations for each respective image. B, benign; M, malignant.

	True H&E-stained histology				Virtual histology			
Image	P1	P2	P3	Consensus	P1	P2	P3	Consensus
1	B	M	B	**B**	B	M	B	**B**
2	M	M	M	**M**	M	M	M	**M**
3	M	M	M	**M**	M	M	M	**M**
4	B	B	B	**B**	B	B	B	**B**
5	B	B	B	**B**	B	B	B	**B**
6	B	B	B	**B**	B	B	B	**B**
7	B	B	B	**B**	B	M	B	**B**
8	B	B	B	**B**	B	B	B	**B**
9	M	M	M	**M**	M	M	M	**M**
10	M	M	M	**M**	M	M	M	**M**
11	B	B	B	**B**	B	B	B	**B**
12	M	M	M	**M**	B	M	M	**M**
13	M	M	M	**M**	M	M	M	**M**
14	B	B	B	**B**	B	B	B	**B**
15	M	M	M	**M**	M	M	M	**M**
16	B	M	M	**M**	B	B	B	**B**
17	M	M	M	**M**	B	M	B	**B**
18	M	M	M	**M**	M	M	M	**M**
19	M	M	M	**M**	M	M	M	**M**
20	M	M	M	**M**	M	M	M	**M**
21	M	M	M	**M**	M	M	M	**M**
22	M	M	M	**M**	M	M	M	**M**
23	B	B	B	**B**	B	B	B	**B**
24	M	M	M	**M**	M	M	M	**M**
25	M	M	M	**M**	M	M	M	**M**
26	M	M	M	**M**	M	M	M	**M**
27	B	B	B	**B**	B	B	B	**B**
28	B	B	B	**B**	B	B	B	**B**
29	B	B	B	**B**	B	B	B	**B**
30	M	M	M	**M**	B	M	B	**B**
31	M	M	M	**M**	M	M	M	**M**
32	M	M	M	**M**	M	M	M	**M**

Table 5. **Deep learning-enabled prostate tissue virtual histology diagnostic concordance study results.** Pathologist diagnostic concordance study results for deep learning-enabled prostate tissue virtual histology images and true-H&E stained counterparts, with a consensus value representing the mode of pathologist (P) interpretations for each respective image. B, benign; M, malignant.

	Pathologist 1			Pathologist 2			Pathologist 3			Mean over Pathologists		
**Image**	**HD**	**ED**	**SQ**	**HD**	**ED**	**SQ**	**HD**	**ED**	**SQ**	**HD**	**ED**	**SQ**
**1 (VS)**	2	2	2	3	2	2	2	1	1	2.33	1.67	1.67
**2 (HS)**	1	1	1	2	3	3	1	1	1	1.33	1.67	1.67
**3 (VS)**	2	2	2	3	2	2	1	1	1	2.00	1.67	1.67
**4 (VS)**	3	3	3	2	2	2	3	3	2	2.67	2.67	2.33
**5 (HS)**	2	2	2	2	3	2	3	3	2	2.33	2.67	2.00
**6 (VS)**	3	3	3	2	2	2	2	2	2	2.33	2.33	2.33
**7 (HS)**	2	3	3	1	2	1	2	3	3	1.67	2.67	2.33
**8 (VS)**	2	2	2	3	1	2	2	2	2	2.33	1.67	2.00
**9 (HS)**	2	3	2	2	1	2	3	3	3	2.33	2.33	2.33
**10 (VS)**	2	1	2	1	3	2	2	1	1	1.67	1.67	1.67
**11 (HS)**	3	2	2	2	1	2	3	3	3	2.67	2.00	2.33
**12 (HS)**	2	3	2	1	3	2	2	3	2	1.67	3.00	2.00
**13 (VS)**	1	2	1	2	3	2	2	3	2	1.67	2.67	1.67
**14 (VS)**	3	2	2	2	1	2	3	3	2	2.67	2.00	2.00
**15 (HS)**	2	2	2	1	1	1	2	2	2	1.67	1.67	1.67
**16 (VS)**	2	3	2	3	3	3	2	2	2	2.33	2.67	2.33
**17 (HS)**	2	2	2	2	2	2	1	2	1	1.67	2.00	1.67
**18 (HS)**	3	3	3	1	3	2	3	3	3	2.33	3.00	2.67
**19 (VS)**	2	3	2	2	3	3	3	2	3	2.33	2.67	2.67
**20 (HS)**	3	2	2	1	2	2	2	2	2	2.00	2.00	2.00
**21 (HS)**	2	2	2	1	1	1	1	1	1	1.33	1.33	1.33
**22 (VS)**	3	2	2	3	3	3	2	2	2	2.67	2.33	2.33
**23 (HS)**	2	2	2	1	1	1	1	2	1	1.33	1.67	1.33
**24 (HS)**	2	2	2	1	2	2	2	3	2	1.67	2.33	2.00
**25 (VS)**	2	3	2	2	3	3	2	2	3	2.00	2.67	2.67
**26 (VS)**	3	3	3	2	1	2	3	3	4	2.67	2.33	3.00
**27 (VS)**	2	3	2	2	3	3	2	2	2	2.00	2.67	2.33
**28 (HS)**	2	3	2	1	2	2	1	2	1	1.33	2.33	1.67
**29 (HS)**	2	2	2	1	1	1	2	2	2	1.67	1.67	1.67
**30 (VS)**	3	3	3	3	2	2	3	3	3	3.00	2.67	2.67
**Mean over VS**	2.33	2.47	2.20	2.33	2.27	2.33	2.27	2.13	2.13	2.31	2.29	2.22
**Mean over HS**	2.13	2.27	2.07	1.33	1.87	1.73	1.93	2.33	1.93	1.80	2.16	1.91
**SEM over VS**	0.16	0.17	0.14	0.16	0.21	0.13	0.15	0.19	0.22	0.10	0.11	0.11
**SEM over HS**	0.13	0.15	0.12	0.13	0.22	0.15	0.21	0.19	0.21	0.11	0.13	0.10

Table 6. Blinded pathologist survey of subjective stain quality. A summary of scores for each image and pathologist rater. VS, deep learning-enabled virtual H&E stain; HS, frozen section H&E-stained histological stain; HD, hematoxylin detail; ED, eosin detail; SQ, overall stain quality; SEM, standard error of the mean. Ratings were provided on the following scale: 1, unacceptable; 2, acceptable; 3, very good quality; 4, perfect stain. Source data are provided as a Source Data file.

References

1. Glaser, A. K. *et al.* Light-sheet microscopy for slide-free non-destructive pathology of large clinical specimens. *Nat. biomedical engineering* **1**, 1–10 (2017).
2. Xie, W. *et al.* Diagnosing 12 prostate needle cores within an hour of biopsy via open-top light-sheet microscopy. *J. Biomed. Opt.* **25**, 126502–126502 (2020).
3. Ragazzi, M. *et al.* Fluorescence confocal microscopy for pathologists. *Mod. Pathol.* **27**, 460–471 (2014).
4. Pérez-Anker, J. *et al.* Basal cell carcinoma characterization using fusion ex vivo confocal microscopy: a promising change in conventional skin histopathology. *Br. J. Dermatol.* **182**, 468–476 (2020).
5. Li, J. *et al.* Biopsy-free in vivo virtual histology of skin using deep learning. *Light. Sci. & Appl.* **10**, 233 (2021).
6. Fereidouni, F. *et al.* Microscopy with ultraviolet surface excitation for rapid slide-free histology. *Nat. biomedical engineering* **1**, 957–966 (2017).
7. Wang, M. *et al.* High-resolution rapid diagnostic imaging of whole prostate biopsies using video-rate fluorescence structured illumination microscopy. *Cancer research* **75**, 4032–4041 (2015).
8. Zhang, Y. *et al.* High-throughput, label-free and slide-free histological imaging by computational microscopy and unsupervised learning. *Adv. Sci.* **9**, 2102358 (2022).
9. Zhou, C. *et al.* Integrated optical coherence tomography and microscopy for ex vivo multiscale evaluation of human breast tissues. *Cancer research* **70**, 10071–10079 (2010).
10. Ha, R. *et al.* Optical coherence tomography: a novel imaging method for post-lumpectomy breast margin assessment—a multi-reader study. *Acad. radiology* **25**, 279–287 (2018).
11. Winetraub, Y. *et al.* Oct2hist: Non-invasive virtual biopsy using optical coherence tomography. *medRxiv* (2021).
12. Patel, K. B. *et al.* High-speed light-sheet microscopy for the in-situ acquisition of volumetric histological images of living tissue. *Nat. Biomed. Eng.* 1–15 (2022).
13. Tao, Y. K. *et al.* Assessment of breast pathologies using nonlinear microscopy. *Proc. Natl. Acad. Sci.* **111**, 15304–15309 (2014).
14. Cahill, L. C. *et al.* Nonlinear microscopy for detection of prostate cancer: analysis of sensitivity and specificity in radical prostatectomies. *Mod. Pathol.* **33**, 916–923 (2020).
15. Sun, Y. *et al.* Real-time three-dimensional histology-like imaging by label-free nonlinear optical microscopy. *Quant. Imaging Medicine Surg.* **10**, 2177 (2020).
16. Wong, T. T. *et al.* Fast label-free multilayered histology-like imaging of human breast cancer by photoacoustic microscopy. *Sci. advances* **3**, e1602168 (2017).
17. Imai, T. *et al.* High-throughput ultraviolet photoacoustic microscopy with multifocal excitation. *J. biomedical optics* **23**, 036007 (2018).
18. Cao, R. *et al.* Label-free intraoperative histology of bone tissue via deep-learning-assisted ultraviolet photoacoustic microscopy. *Nat. Biomed. Eng.* **7**, 124–134 (2023).
19. Kang, L., Li, X., Zhang, Y. & Wong, T. T. Deep learning enables ultraviolet photoacoustic microscopy based histological imaging with near real-time virtual staining. *Photoacoustics* **25**, 100308 (2022).
20. Soltani, S. *et al.* Prostate cancer histopathology using label-free multispectral deep-uv microscopy quantifies phenotypes of tumor aggressiveness and enables multiple diagnostic virtual stains. *Sci. Reports* **12**, 9329 (2022).
21. Orringer, D. A. *et al.* Rapid intraoperative histology of unprocessed surgical specimens via fibre-laser-based stimulated raman scattering microscopy. *Nat. biomedical engineering* **1**, 1–13 (2017).
22. Hollon, T. C. *et al.* Near real-time intraoperative brain tumor diagnosis using stimulated raman histology and deep neural networks. *Nat. medicine* **26**, 52–58 (2020).
23. Ecclestone, B. R. *et al.* Label-free complete absorption microscopy using second generation photoacoustic remote sensing. *Sci. Reports* **12**, 8464 (2022).
24. Boktor, M. *et al.* Virtual histological staining of label-free total absorption photoacoustic remote sensing (ta-pars). *arXiv preprint arXiv:2203.02584* (2022).
25. Boktor, M. *et al.* Multi-channel feature extraction for virtual histological staining of photon absorption remote sensing images. *arXiv preprint arXiv:2307.01824* (2023).

26. Tweel, J. E. *et al.* Virtual histology with photon absorption remote sensing using a cycle-consistent generative adversarial network with weakly registered pairs. *arXiv preprint arXiv:2306.08583* (2023).

27. Tweel, J. E. D. *et al.* Photon absorption remote sensing imaging of breast needle core biopsies is diagnostically equivalent to gold standard h&e histologic assessment. *medRxiv* 2023–08 (2023).

28. Zhang, Y. *et al.* Digital synthesis of histological stains using micro-structured and multiplexed virtual staining of label-free tissue. *Light. Sci. & Appl.* **9**, 78 (2020).

29. Bai, B. *et al.* Label-free virtual her2 immunohistochemical staining of breast tissue using deep learning. *BME Front.* **2022** (2022).

30. Borhani, N., Bower, A. J., Boppart, S. A. & Psaltis, D. Digital staining through the application of deep neural networks to multi-modal multi-photon microscopy. *Biomed. optics express* **10**, 1339–1350 (2019).

31. Pradhan, P. *et al.* Computational tissue staining of non-linear multimodal imaging using supervised and unsupervised deep learning. *Biomed. Opt. Express* **12**, 2280–2298 (2021).

32. Zhang, G. *et al.* Image-to-images translation for multiple virtual histological staining of unlabeled human carotid atherosclerotic tissue. *Mol. Imaging Biol.* 1–11 (2022).

33. Rivenson, Y. *et al.* Phasestain: the digital staining of label-free quantitative phase microscopy images using deep learning. *Light. Sci. & Appl.* **8**, 23 (2019).

34. Nygate, Y. N. *et al.* Holographic virtual staining of individual biological cells. *Proc. Natl. Acad. Sci.* **117**, 9223–9231 (2020).

35. Kaza, N., Ojaghi, A. & Robles, F. E. Virtual staining, segmentation, and classification of blood smears for label-free hematology analysis. *BME Front.* **2022** (2022).

36. Chen, Z., Yu, W., Wong, I. H. & Wong, T. T. Deep-learning-assisted microscopy with ultraviolet surface excitation for rapid slide-free histological imaging. *Biomed. Opt. Express* **12**, 5920–5938 (2021).
