# Supplementary material for: Deep learning-enabled realistic virtual histology with ultraviolet photoacoustic remote sensing microscopy
Source: Nat Commun. 2023 Sep 25;14:5967. doi: 10.1038/s41467-023-41574-2 (PMC10519961; doi:10.1038/s41467-023-41574-2)
Supplement: Supplementary file 2 — Reporting Summary [file 41467_2023_41574_MOESM2_ESM.pdf]

nature portfolio

Corresponding author(s): Roger Zemp

Last updated by author(s): Aug 31, 2023

Reporting Summary

Nature Portfolio wishes to improve the reproducibility of the work that we publish. This form provides structure for consistency and transparency in reporting. For further information on Nature Portfolio policies, see our Editorial Policies and the Editorial Policy Checklist.

Statistics

For all statistical analyses, confirm that the following items are present in the figure legend, table legend, main text, or Methods section.

n/a	Confirmed	
☐	☒	The exact sample size (*n*) for each experimental group/condition, given as a discrete number and unit of measurement
☐	☒	A statement on whether measurements were taken from distinct samples or whether the same sample was measured repeatedly
☐	☒	The statistical test(s) used AND whether they are one- or two-sided<br>*Only common tests should be described solely by name; describe more complex techniques in the Methods section.*
☒	☐	A description of all covariates tested
☐	☒	A description of any assumptions or corrections, such as tests of normality and adjustment for multiple comparisons
☐	☒	A full description of the statistical parameters including central tendency (e.g. means) or other basic estimates (e.g. regression coefficient) AND variation (e.g. standard deviation) or associated estimates of uncertainty (e.g. confidence intervals)
☐	☒	For null hypothesis testing, the test statistic (e.g. *F*, *t*, *r*) with confidence intervals, effect sizes, degrees of freedom and *P* value noted<br>*Give P values as exact values whenever suitable.*
☒	☐	For Bayesian analysis, information on the choice of priors and Markov chain Monte Carlo settings
☒	☐	For hierarchical and complex designs, identification of the appropriate level for tests and full reporting of outcomes
☐	☒	Estimates of effect sizes (e.g. Cohen's *d*, Pearson's *r*), indicating how they were calculated

Our web collection on statistics for biologists contains articles on many of the points above.

Software and code

Policy information about availability of computer code

Data collection	Virtual histology image data was collected using a custom laboratory bench-top ultraviolet photoacoustic remote sensing and ultraviolet scattering microscope, with acquisition performed using motorized scanning stages controlled by MATLAB GUI software. Control of data acquisition was performed using custom, proprietary C++ programs. Brightfield H&E-stained images were captured using a Leica Microsystems whole-slide scanning system and associated Aperio ImageScope software.
Data analysis	Reconstruction of UV-PARS and UV scattering channels from raw data, preparation of training data sets, image registration, analysis of image similarity, and graphical data analysis were all performed using custom MATLAB scripts, which are available from the authors on request within 3 weeks, where not proprietary. The deep learning model was trained based on a publicly-available implementation of the CycleGAN algorithm, which can be accessed in the following GitHub repository: https://junyanz.github.io/CycleGAN/. Segmentation and analysis of cell nuclei morphology was performed using the open-source CellProfiler software (https://cellprofiler.org).

For manuscripts utilizing custom algorithms or software that are central to the research but not yet described in published literature, software must be made available to editors and reviewers. We strongly encourage code deposition in a community repository (e.g. GitHub). See the Nature Portfolio guidelines for submitting code & software for further information.

Data

Policy information about availability of data

All manuscripts must include a data availability statement. This statement should provide the following information, where applicable:

- Accession codes, unique identifiers, or web links for publicly available datasets
- A description of any restrictions on data availability
- For clinical datasets or third party data, please ensure that the statement adheres to our policy

The main data supporting the findings of this study are available within the paper and its Supplementary Information. The training, test, and validation datasets for the breast tissue and prostate tissue staining models are available at https://doi.org/10.5281/zenodo.7981075. Due to size considerations, raw data will be made available upon reasonable request.

Human research participants

Policy information about studies involving human research participants and Sex and Gender in Research.

Reporting on sex and gender	Prostate tissues were obtained exclusively from males who have had a radical prostatectomy procedure.<br><br>Breast tissues were obtained exclusively from female subjects due to the relatively lower incidence of breast cancer in the male population.
Population characteristics	Breast tissue imaging: malignant specimens from patients undergoing a mastectomy or lumpectomy procedure with a confirmed invasive ductal carcinoma or ductal carcinoma in-situ diagnosis, and benign specimens discarded from reduction mammoplasty procedures. Samples representative of benign breast histology were obtained from a reduction mammoplasty procedure, where the tissue would have otherwise been discarded. Breast tissue specimens represented 9 female subjects ranging in age from 35-69.<br><br>Prostate tissue imaging: specimens obtained from radical prostatectomy procedures. Prostate tissue specimens included cases from 7 male patients ranging in age from 55-71.
Recruitment	All tissue samples were obtained from pathology laboratories after cases were closed and tissues were otherwise flagged for disposal. All samples were de-identified of patient-related information and pathology reports were blinded.<br><br>Histological frozen sections of human breast tissues are not regularly collected at this institution, and therefore anonymized samples were obtained from the OriGene tissue bank.
Ethics oversight	Health Research Ethics Board of Alberta (HREBA.CC-20-0145)

Note that full information on the approval of the study protocol must also be provided in the manuscript.

Field-specific reporting

Please select the one below that is the best fit for your research. If you are not sure, read the appropriate sections before making your selection.

☒ Life sciences ☐ Behavioural & social sciences ☐ Ecological, evolutionary & environmental sciences

For a reference copy of the document with all sections, see nature.com/documents/nr-reporting-summary-flat.pdf

Life sciences study design

All studies must disclose on these points even when the disclosure is negative.

Sample size	Training data consisted of a set of 12000 image patches of human breast virtual histology and 12000 true human breast histology patches, with equal proportions of benign and malignant examples. Training data consisted of a set of 16000 image patches of human prostate virtual histology and 16000 true human prostate histology patches, with equal proportions of benign and malignant examples. The mouse liver and kidney tissue models were further trained by transfer learning with sets of 200-300 512x512 patches. Quantitative metric validation was performed using 1921 image patch pairs from one-to-one matched true histology and virtual histology, using new examples unseen in the training. The diagnostic concordance study featured 24 and 32 pairs of matching true and virtual histology images for breast and prostate, respectively. The blinded stain quality survey of pathologists used 15 virtual histology images and 15 random frozen section images provided by a tissue bank. This sample size may be justified a posteriori, as a statistically significant result was obtained.
Data exclusions	Training datasets were constructed computationally by randomly cropping image patches from larger field of view images. Patches were manually excluded only in cases where the 400x magnification whole-slide scan was out of focus, dust particles were present in the field of view, or the patch contained only glass slide/coverslip and not tissue.
Replication	Deep learning-enabled virtual histology imaging performance was evaluated on multiple tissue specimens of different types, including human

	breast, human prostate, mouse kidney, and mouse liver. All images depict single scans of single samples to demonstrate proof of concept, though all data sets are representative of multiple (at least three) repeated experiments where the same histological features were consistently resolved.
Randomization	Virtual images were obtained by scanning random regions of unstained tissues without microscopic guidance by other imaging modalities. True brightfield H&E-stained histology training datasets were obtained by randomly cropping patches from whole-slide images. A random selection of frozen sections was provided by the OriGene tissue bank, with disease states comparable to tissues imaged with virtual histology being the only criterion for inclusion. Allocation of experimental groups and control of covariates was not relevant to this study, with randomization serving primarily to achieve representation of histological variations in imaging data sets.
Blinding	The trained deep learning model was tested on new tissue images which were not included in the training or validation datasets. For the diagnostic concordance study, pathologists were blinded to whether the presented images were true brightfield H&E-stained histology or virtual histology. The pathologists identified benign vs. malignant tissues while blinded to pathology reports associated with the specimens, and the pathologists of record who originally signed-out each case were not included in the panel.

Reporting for specific materials, systems and methods

We require information from authors about some types of materials, experimental systems and methods used in many studies. Here, indicate whether each material, system or method listed is relevant to your study. If you are not sure if a list item applies to your research, read the appropriate section before selecting a response.

Materials & experimental systems

n/a	Involved in the study
☒	☐ Antibodies
☒	☐ Eukaryotic cell lines
☒	☐ Palaeontology and archaeology
☐	☒ Animals and other organisms
☒	☐ Clinical data
☒	☐ Dual use research of concern

Methods

n/a	Involved in the study
☒	☐ ChIP-seq
☒	☐ Flow cytometry
☒	☐ MRI-based neuroimaging

Animals and other research organisms

Policy information about studies involving animals; ARRIVE guidelines recommended for reporting animal research, and Sex and Gender in Research

Laboratory animals	Swiss Webster mouse (Crl:CFW(SW), Charles River Laboratories), Age: 1-3 months, Sex: Female. Housing conditions for mice were maintained as follows: 12:12 hours light:dark cycles, 20-26 degC ambient temperature, and a 50-70 % relative ambient humidity.
Wild animals	No wild animals were involved in this study.
Reporting on sex	Only one sex was utilized in this report. Sex was not considered in the study design as the primary focus of this work was proof of concept tissue histological imaging.
Field-collected samples	No field collected samples were used in this study.
Ethics oversight	All animal experiments were conducted in accordance with ethics protocols approved by the University of Alberta Animal Care and Use Committee (AUP00001170).

Note that full information on the approval of the study protocol must also be provided in the manuscript.
